# Trends in outcomes following COVID-19 symptom onset in Milan: a cohort study

**DOI:** 10.1136/bmjopen-2021-054859

**Published:** 2022-03-23

**Authors:** Christopher H Jackson, Francesca Grosso, Kevin Kunzmann, Alice Corbella, Maria Gramegna, Marcello Tirani, Silvana Castaldi, Danilo Cereda, Daniela De Angelis, Anne Presanis

**Affiliations:** 1 MRC Biostatistics Unit, University of Cambridge, Cambridge, UK; 2 Postgraduate School of Public Health, Department of Biomedical Sciences for Health, University of Milan, Milan, Italy; 3 Department of Statistics, University of Warwick, Coventry, UK; 4 Welfare General Directorate, Regione Lombardia, Milan, Italy; 5 Post-graduate School of Hygiene and Preventive Medicine, University of Milan, Milan, Italy

**Keywords:** COVID-19, epidemiology, public health

## Abstract

**Background:**

For people with symptomatic COVID-19, the relative risks of hospital admission, death without hospital admission and recovery without admission, and the times to those events, are not well understood. We describe how these quantities varied with individual characteristics, and through the first wave of the pandemic, in Milan, Italy.

**Methods:**

A cohort study of 27 598 people with known COVID-19 symptom onset date in Milan, Italy, testing positive between February and June 2020 and followed up until 17 July 2020. The probabilities of different events, and the times to events, were estimated using a mixture multistate model.

**Results:**

The risk of death without hospital admission was higher in March and April (for non-care home residents, 6%–8% compared with 2%–3% in other months) and substantially higher for care home residents (22%–29% in March). For all groups, the probabilities of hospitalisation decreased from February to June. The probabilities of hospitalisation also increased with age, and were higher for men, substantially lower for healthcare workers and care home residents, and higher for people with comorbidities. Times to hospitalisation and confirmed recovery also decreased throughout the first wave. Combining these results with our previously developed model for events following hospitalisation, the overall symptomatic case fatality risk was 15.8% (15.4%–16.2%).

**Conclusions:**

The highest risks of death before hospital admission coincided with periods of severe burden on the healthcare system in Lombardy. Outcomes for care home residents were particularly poor. Outcomes improved as the first wave waned, community healthcare resources were reinforced and testing became more widely available.

**Strengths and limitations of this study**

- An analysis of a database of all COVID-19 cases in Milan between February and June 2020.
- Uses multistate modelling to estimate relative risks of hospital admission, death without admission and recovery without admission, jointly with the times to those events, for different groups of people diagnosed with COVID-19 in the community.
- The model for events following onset is combined with a previous model for analysis of outcomes following hospital admission, to enable predictions of final outcomes for people of different groups diagnosed in the community.
- Changes through time in outcomes are hard to attribute confidently to causal effects of changing healthcare burden and improvements in healthcare resources, due to possible selection biases from changes through time in testing availability and policy.

## Introduction

There is now an extensive literature on the risks of COVID-19 hospitalisation and fatality.

The majority of studies of mortality have either estimated the risk in hospital cohorts^1–7^ or in specific settings such as care homes^8–10^ and a cruise ship.^11^ There have been fewer studies of mortality among the general population. Poletti *et al*^12^ estimated population infection fatality risks using contacts of confirmed cases, while Salje *et al*^13^ estimated infection fatality risks from a transmission model informed by multiple sources. Some have estimated population symptomatic case fatality risks (sCFRs) from aggregate time series data with limited information on risk factors.^14–16^ Bhaskaran *et al*^17^ conducted a large population study of COVID-19 mortality, focusing on relative risks between subgroups rather than absolute risks of mortality, and without considering hospital admission. There have been many studies of COVID-19 hospitalisation from individual-level data, many focusing on specific risk factors, for example, for healthcare workers,^18^ by ethnicity,^19^ by virus variant,^20–23^ by frailty^24^ or for more general risk factors.^25^ Very few, however, have jointly estimated the competing risks of multiple outcomes, such as hospital or critical care admission, death, hospital discharge and recovery, and how these varied over time^3 6 26^ and with individual risk factors.^7^ While those studies concentrated on post-hospital outcomes, our study examines the competing risks of hospital admission, death and recovery for people with symptomatic COVID-19 outside of hospitals, for the population of Milan.

In Lombardy, the first person in Italy was diagnosed with COVID-19 in mid-February 2020. In Milan, confirmed cases rose from 403 on the 27 February 2020 to 22 264 at the first wave peak on 20 March 2020. This abrupt rise, not only in Milan but across Lombardy, put a large strain on the region’s healthcare system.^27^ This is likely to have impacted also on outcomes for people with COVID-19 in the community, by which we mean outside of secondary healthcare. The regional health system of Lombardy differs substantially from those of other Italian regions, being characterised by a strict separation of primary/community and secondary healthcare provision by the Local Health Authority (LHA) in each district in the region. At the beginning of the pandemic, each LHA’s Infectious Disease Territorial Unit was overwhelmed by the number of notifications of COVID-19 cases. The LHAs organised extra staff, laboratory and information technology resources to speed up contact tracing and management of cases. From 11 March, general practices restricted their in-person services, and from 30 March, care home facilities were closed to visitors. Further details of the regional system and its response to the pandemic are given in the online supplemental table 1.

10.1136/bmjopen-2021-054859.supp1 Supplementary data

An integrated database was created to collate detailed pseudo-anonymised data from the LHAs’ combined symptom and laboratory surveillance, and from hospitals, on each PCR-confirmed case of COVID-19 in Lombardy. These data have been described in full elsewhere,^6 7 28^ but briefly, comprise dates of all relevant events from symptom onset through testing to final outcomes of either recovery, discharge or death. The information on symptom onset is thought to be most reliable from the Milan LHA. From these data, we present a retrospective cohort study among symptomatic confirmed COVID-19 cases in Milan to estimate the burden of COVID-19 in the community. We use a mixture multistate model^29 30^ to jointly estimate the competing risks of death before hospitalisation, hospital admission and recovery without hospital admission, as well as the times from symptom onset to each of these events. We assess how these risks and times changed over the months of the first wave of infection in Milan, and we combine our estimates of community outcomes with our previous estimates of within-hospital outcomes^6 7^ to estimate the symptomatic confirmed case fatality risk, averaging over deaths in the community, in hospital before critical care and in critical care. For this region and population, these provide stronger information than the crude case fatality rates reported by Riccardo *et al*.^31^

## Methods

### Study participants

The dataset represents all individuals in Lombardy diagnosed with COVID-19 during the first wave from February to June 2020,^6 7 28^ excluding those admitted to hospital whose symptom onset date was after their hospital admission date, diagnosed after hospital admission. As extracted on 5 August 2020, it includes 29 347 individuals in the Milan area with confirmed COVID-19 who first tested positive from 1 February 2020 to 30 June 2020. The dataset records age, gender, comorbidities, symptoms, whether the individual is a healthcare worker or care home resident, and whether they were hospitalised. Dates of symptom onset, positive laboratory test, hospital admission, confirmed recovery and death are recorded. Confirmed recovery is defined as having had two consecutive negative PCR tests and no more symptoms—note that the time to confirmed recovery will be longer than the (unknown) time to clinical recovery. Events after hospital admission are excluded from the main analysis of (first) outcomes following symptom onset. After excluding records with inconsistent or invalid dates, 27 598 individuals remain in the dataset.

### Multistate model

To make use of the information on relative risks of events from all individuals, including the 7% who had none of the events recoded in the data period, a multistate model is implemented. This represents how a person transitions from an ‘initial’ state representing current COVID-19 infection, to three alternative states, representing hospital admission, confirmed recovery from infection without having been hospitalised or death without having been hospitalised (online supplemental figure 1). The outcomes of interest are which of these three alternative events is experienced by a person diagnosed with COVID-19 outside hospital, and the time from COVID-19 symptom onset until that event happens.

The multistate model is implemented as a ‘mixture model’,^29 30^ using the *flexsurv* R package,^32^ combining multinomial or binomial logistic regression of probabilities of different events following onset with parametric time-to-event analyses for the time taken from onset to each event, given that the event occurred. For the individuals where no outcome was recorded, it is assumed that no event had happened either by the closing date of the data, 19 July, or at 60 days after onset if this point is before 19 July. This maximum censoring time of 60 days is imposed since many individuals with onset in February–March had not had an event recorded by July, and we assume that it is implausible that none of these events would have occurred over such a long follow-up. An alternative analysis is considered, in which all individuals with missing outcomes are excluded.

We consider models that include the following covariates: gender, age group (three categories: age 45 and below, age 46–65, and age 66 and above), month of onset (five categories: February, March, April, May and June), whether the individual is a healthcare worker, or had any comorbidities, or is a care home resident. Covariates were considered to affect both the probability of each event occurring (through multinomial logistic regression) and the expected time to the event (through a generalised gamma accelerated failure time model). Full details of the regression model specification are given in the online supplemental appendix. Point estimates and 95% CIs are reported for each event probability for a person with each combination of characteristics, as well as for the median and the 95% quantile interval (between the 2.5% and 97.5% quantiles) of each time-to-event distribution. The CIs represent parameter uncertainty, whereas the median and quantile intervals represent heterogeneity across individuals in the time-to-event distributions.

The multistate model in the online supplemental figure 1 can be combined with the within-hospital multistate model of Grosso *et al* and Presanis *et al*^6 7^ applied to the Milan data, to obtain an overall estimate of the sCFR, averaged over deaths in the community and in hospital. Results from two combined models are presented: one with no covariates; and one where the mixture probabilities and the location parameters of the time-to-event distributions are regressed on age, gender and month of symptom onset.

### Patient and public involvement

Although patients were not directly involved in the study design, the experiences of clinicians and public health officials interacting with patients were crucial to the design of the data collection.

## Results

### Dataset description

The 27 598 people in the cohort had a median age of 64 years (IQR 48–82), 45% were male and 55% were female, 13% were healthcare workers, 21% were care home residents and 47% had at least one comorbidity. Overall, 43% of people were admitted to hospital, while 5% died without being admitted, 45% recovered without being admitted and 7% had none of these events recorded. Online supplemental table 2 summarises how many people in each subgroup of interest were observed to have each of these potential events.

### Probabilities of hospital admission and death

As estimates were substantively similar whether or not missing outcomes were assumed to represent censoring (online supplemental tables 3–12 and online supplemental figures 2 and 3), we report estimates from the model assuming censoring. The selected models for the odds of admission and death are detailed in the online supplemental tables 13–17. The main effects of all covariates and several two-way interactions are included in both models (except that no healthcare workers died before hospital admission, therefore this predictor was not included in the model for death without admission).

**Figure 1:**
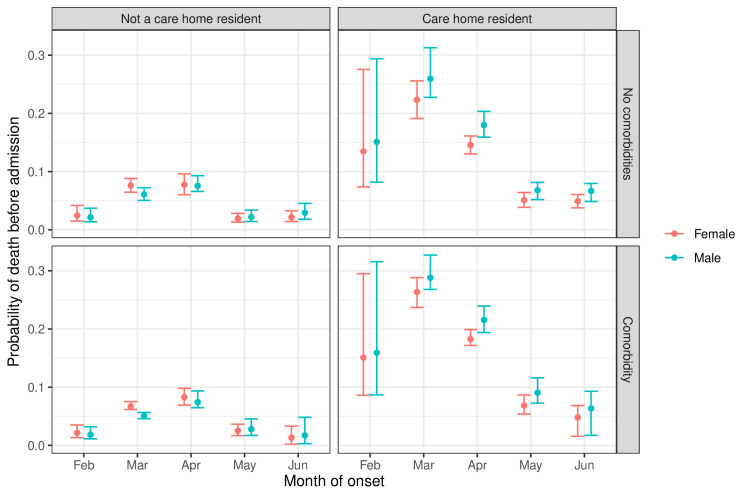
Probability of death before hospital admission by month of COVID-19 onset, gender, and whether a person is a care home resident or had comorbidities. Only considers people over 65 years of age who were not healthcare workers. Estimates and 95% CIs.

There were only 28 deaths (<1%) observed from people under 65 years, so we report mortality results for the 65+ years age group only. The probability of death without hospital admission (figure 1, online supplemental tables 3 and 4) was highest in March and April: 6% (95% CI 5% to 7%) and 8% (6% to 9%), respectively, for men and women over 65 years in the baseline risk group in March, compared with 2%–3% in other months. The probability of death without hospital admission was highest for care home residents: 22% (19% to 25%) for women and 26% (23% to 30%) for men without comorbidities in March; and even higher in care home residents with comorbidities: 26% (25% to 29%) for women and 29% (26% to 32%) for men in March. The effect of gender and comorbidities on this risk was only significant for those in care homes. While care home residents were older on average (mean age 85 years, compared with 58 years), this mortality was higher for care home residents within all age groups, even with a more detailed age grouping (online supplemental table 18).

**Figure 2:**
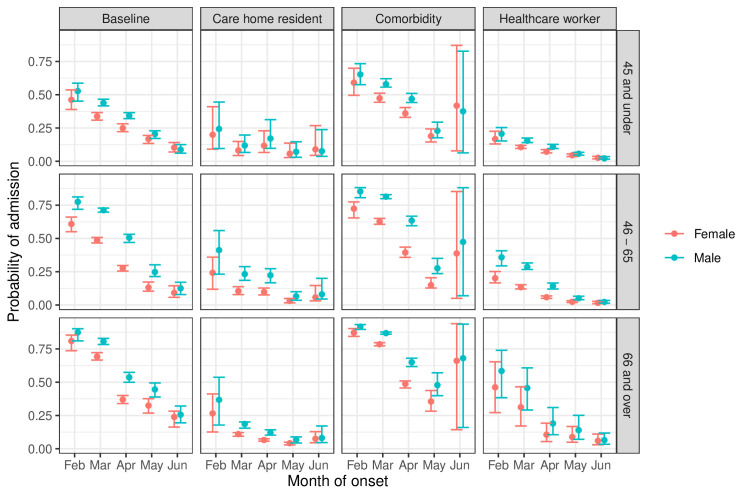
Probability of hospital admission following COVID-19 onset, by month of onset, age group and gender, for care home residents, people with comorbidities and healthcare workers, compared with a baseline individual with none of these risk factors. Estimates and 95% CIs.

The probabilities of hospitalisation for various subgroups are compared in figure 2. These risks decreased through the first wave in all groups, for example, from 87% (85% to 90%) in February to 26% (18% to 33%) in June for men who are aged over 65 years in the baseline risk group (neither care home residents nor healthcare workers, with no comorbidities) and from 81% (78% to 86%) to 24% (17% to 32%) in women in the same group. Probabilities of hospitalisation were higher for men (81%, 79% to 82%) and people with comorbidities (79%, 77% to 80%) and lower for younger people (49%, 46% to 50%, ages 45–65 years), healthcare workers (31%, 20% to 45%) and care home residents (11%, 9% to 12%), compared with 69% (66% to 71%) for a baseline group defined by women aged 65+ years who were neither care home residents nor healthcare workers, with no comorbidities, and onset in March. The absolute differences in probabilities of hospital admission between men and women, and between those with and without comorbidities, appear largest in February–April, when hospitals were most stretched, reducing in May–June.

Online supplemental tables 3–6 list the estimated probabilities of all events, by all subgroups, from models with and without the censored data.

### Times to events

Figure 3 summarises estimates of the times to admission, death and recovery in the community. Most notably, times to admission and confirmed recovery became shorter and less variable from February to June. These results are fully detailed in the online supplemental figures 4–11 and online supplemental tables 7–12.

**Figure 3:**
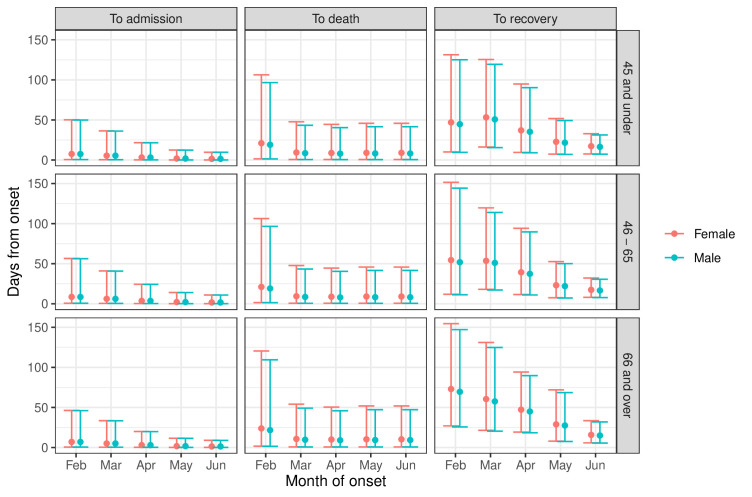
Times from COVID-19 onset to hospital admission, to death before hospital admission and to recovery before hospital admission (median and range containing 95% of individuals), by month of onset, age and gender, for people without comorbidities who were neither care home residents nor healthcare workers. Estimates and intervals including 95% of individuals.

### Combined hospital and community models

**Figure 4:**
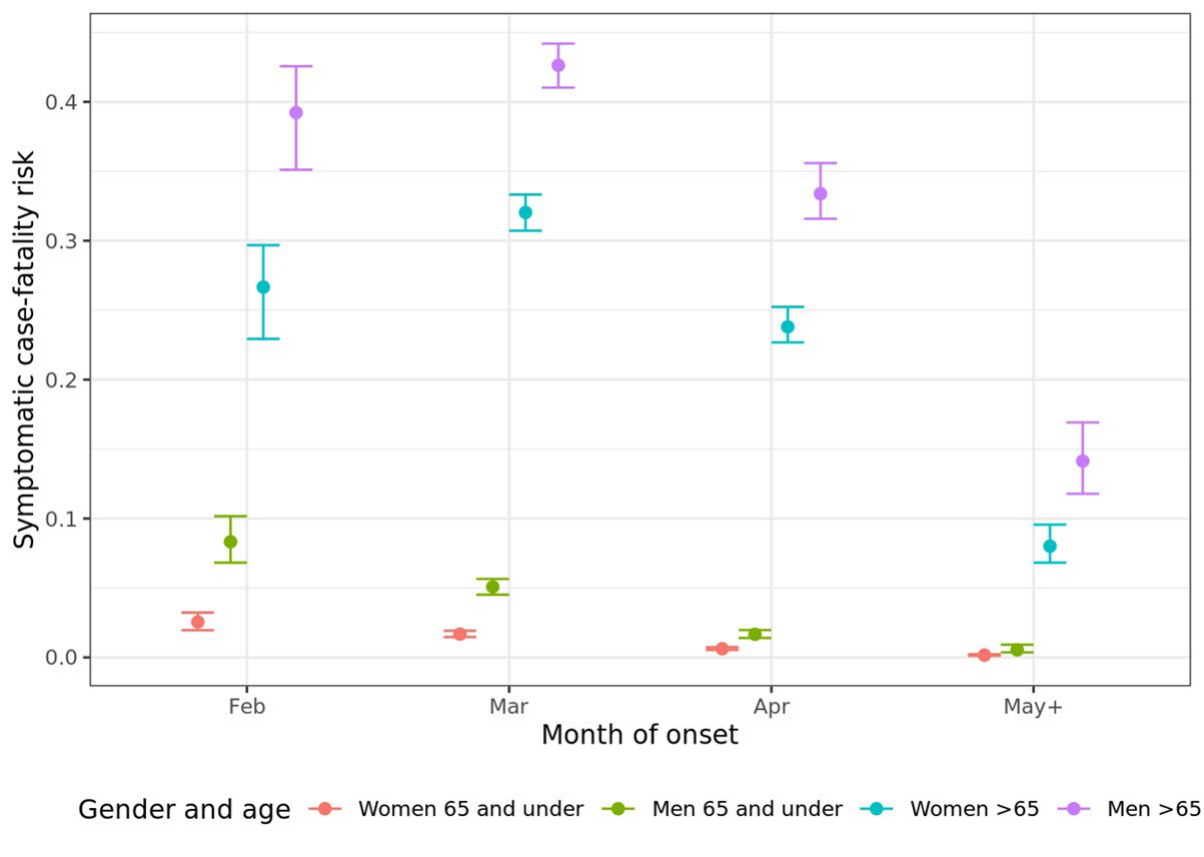
Estimated symptomatic case fatality risk with 95% CIs, by gender, age and month of symptom onset.

Combining the hospital model of Boëlle *et al* and Poletti *et al*^3 12^ with the model of events following a symptom onset presented here, we obtain an overall estimate of the sCFR (averaged over deaths in hospital and in the community, and without accounting for any covariates) of 15.8% (95% CI 15.4% to 16.2%). The online supplemental figures 12 and 13 further describe the probabilities of following each potential pathway through the events of onset, admission, intensive care unit (ICU) admission and death or recovery, under the model without covariates. The estimates of sCFR by gender, age group and calendar month of symptom onset are compared in figure 4. Older age and being male are associated with higher sCFR, while the sCFR has reduced over time from peaks in February and March, respectively, for the younger and older age groups.

**Figure 5:**
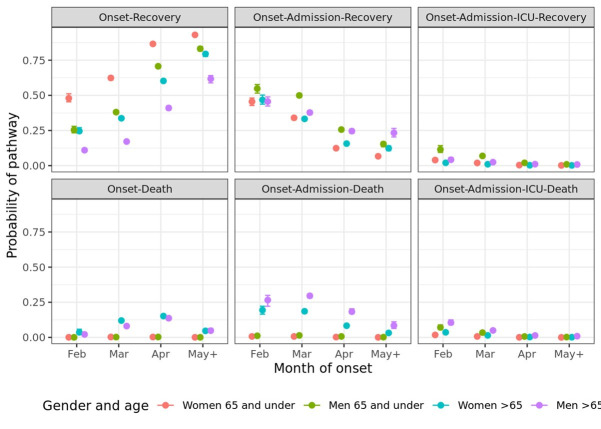
Estimated probability (95% CI) of each pathway defined by sequences of different events, by gender, age and calendar month of symptom onset.

The probabilities of taking each pathway, by gender, age group and month are displayed in figure 5. Again, older age and being male are associated with the more severe pathways. The proportion of people who experience the pathway onset-recovery increases dramatically with calendar month, while the probability of taking a pathway that ends in death decreases with month of onset.

Without taking covariates into account, the median time to death from symptom onset, averaged over pathway, is 14.5 days (95% CI 13.3 to 14.8), but there is substantial variation between individuals, with a 95% quantile interval of 1.6–65.0 days. The distribution of time to confirmed recovery from symptom onset, averaged over pathway, is also highly heterogeneous, with median 34.5 days (95% CI 33.9 to 35.3) and 95% of individuals having times between 4.6 and 107 days.

Further summaries of the distributions of times from symptom onset to final events, overall and by pathway, are given in the online supplemental figures 14 and 15 and online supplemental table 19.

## Discussion

Our analysis has resulted in three key findings concerning risks of severe events in the community following symptom onset among confirmed COVID-19 cases in Milan: (1) groups at elevated risk of death without hospital admission include older age groups, men, care home residents and those with comorbidities, whereas those at higher risk of hospital admission include older age groups, men and those with comorbidities, with care home residents and healthcare workers having substantially lower risk than others; (2) risks of hospital admission and death without hospital admission peaked early in the first wave; (3) these risks steadily decreased with month of symptom onset after their peak.

The first result confirms previous findings of which groups of people with COVID-19 are most at risk of severe events.^1–7 33–35^ In particular, as in Presanis *et al*,^7^ we see a possible protective effect in healthcare workers compared with the general population, who are estimated to be at lower risk of both hospital admission and death without hospitalisation. The previous findings have, however, concentrated on ICU and mortality risk among hospitalised patients, whereas a strength of our analysis is the availability of data allowing us to assess risks following symptom onset in the community.

Hospital admission risk peaked for cases with onset in February, while non-hospital death risk peaked for cases with onset in March for care home residents, and April for all others, while the overall sCFR peaked in February and March. These results suggest that the epidemic spread initially in the general population from February, then spread to care homes only from March and April onwards.^7 36^ The highest risk months coincided with the peak of the first wave in Lombardy, when a great amount of strain was placed on the regional health system. Both primary and secondary healthcare were affected, these services being intertwined in complex dynamics. We surmise that in these months, the sudden exponential growth in the number of severely symptomatic cases per day slipped out of control of the community healthcare in the region, with hospitals overwhelmed and therefore too few beds available for admission. While the hospital burden plausibly affected the prehospital death risk, the effect of hospital burden on rates of admission is harder to predict, since at times of maximum capacity, the rates of admission cannot exceed a certain amount.

Following these peak months, risks of severe events steadily declined. The decreasing trend in probabilities of hospital admission among all groups and increasing probability of confirmed recovery without needing hospital care may reflect a combination of factors. The LHAs’ roles in the pandemic response may have been important. With the increased resources assigned to these providers by Regione Lombardia over the first few months, as well as the decreasing number of confirmed cases as the first wave waned, it has been noted that the Lombardy health system had greater ability to diagnose and treat patients with COVID-19.^37^ This improved ability included greater awareness of general practitioners in treating patients; increased use of pulse oximeters to anticipate disease diagnosis and oxygen need; and greater efficiency of the system due to fewer cases. These improvements may have resulted in the ability to treat a greater proportion of cases outside hospital, resulting in lower hospital burden and better outcomes for the population. However, this interpretation should be taken with some caution: another factor in the decreasing probability of hospital admission may be changes in testing availability and policy over time. A limitation is not being able to account for such changes in our analysis. In the start of the pandemic, the scarcity of swab tests may have resulted in prioritisation of people with severe symptoms for testing. With time, testing became available to a wider range of people, with less severe or no symptoms, increasing the detection rate of positive cases, broadening the denominator for and therefore decreasing the probability of hospital admission. This last hypothesis is also supported by how little the estimated times from onset to death without hospital admission have changed over time (figure 3): indicating perhaps less improvement in treatment interventions at home and in care homes for the most fragile non-hospitalised people, who died within the same interval regardless of month.

As noted above, care home residents were at a very high estimated risk of death without admission and low risk of hospital admission following COVID-19 onset in March–April. Furthermore, as estimated by Presanis *et al*,^7^ these people also had a very high risk of dying in hospital without ICU admission. In contrast, for the general population, probabilities of hospital admission were highest during the first months of the pandemic, and as others have noted, hospitals were overloaded and ambulance availability was limited in this period.^38^ We can therefore speculate that clinicians may have had to triage, not admitting already very frail care home residents. For those care home residents who were admitted, we estimated longer times from symptom onset to admission than other patients. Similar findings were described by Boëlle *et al*.^3^ The estimated lower risk of hospital admission for care home residents may also be an artefact of greater testing in this population, leading to a higher proportion of cases detected being mild ones.

To obtain estimates of absolute risks from censored data, for combinations of risk factors, we used statistical models with parametric assumptions. While the substantive findings are not affected, the exact values of some of our risk estimates may be sensitive to these assumptions for particular combinations of risk factors for which the data were weak, as detailed in the online supplemental appendix.

The trends we have estimated in the risks of outcomes following symptom onset may also be affected by what has been termed ‘epidemic phase bias’.^39^ This occurs because when the epidemic is growing, a greater proportion of people infected will be recent infections, and when the epidemic is waning, a greater proportion will be in the later phase of infection. This affects studies of outcomes following symptom onset (or other proxy events for the unobservable infection time), because cases with onset in the growing phase of the epidemic tend to be more recent infections. Therefore, since cases with shorter times from infection to onset may be more severe cases, we may overestimate risks of hospitalisation or death at times of increasing incidence and underestimate them when incidence is decreasing. The estimated trends over time in risk we obtain may therefore be partially explained by epidemic phase bias. However, using symptom onset date as a proxy for the infection time, as we did, should be less susceptible to this bias compared with using the date of the positive test, being generally closer to the date of infection.

Despite the constraints of interpretation due to testing availability and policy and its change over time, we have nevertheless illustrated important estimates of sCFR and risks of hospital admission and death in the community, and their changes through time. While our study is based on a population without immunity, infected with the original strain of SARS-CoV-2, our estimates still contribute to the overall picture of how outcomes changed through the pandemic, and might still be used in models that combine different sources of evidence on absolute and relative risks, to inform future policymaking.

## Data Availability

Data are not publicly available. This study is based on a database maintained by the Prevention Unit of the General Directorate of Welfare of Regione Lombardia. The authors can forward inquiries relating to use of this database.
