# Supplementary material for: Trends in outcomes following COVID-19 symptom onset in Milan: a cohort study
Source: BMJ Open. 2022 Mar 23;12(3):e054859. doi: 10.1136/bmjopen-2021-054859 (PMC8948075; doi:10.1136/bmjopen-2021-054859)
Supplement: Supplementary data [file bmjopen-2021-054859supp001.pdf]

Appendix

Lombardy regional healthcare system

The regional health system of Lombardy is quite different from those of other Italian regions and is characterised by a strict separation of secondary healthcare provision, performed by state or private hospitals, from primary healthcare and public health in the community, administered by Local Health Authorities (LHA). Regione Lombardia is geographically divided into 8 departments, each with a LHA coordinated and supported by the General Directorate of Welfare of Regione Lombardia. A LHA plans and organises the specific healthcare needs of the population living in its district, including managing the national health system's budget for both private and state hospitals (Cereda et al. 2020). Agreements are made between each LHA and Territorial Health Social Trusts, hospitals and General Practitioners (GPs), with regard to the health services that are needed by the population. Each LHA includes several departments, including: Hygiene and Preventive Care; Primary Care; Programming and Accreditation and Regulation of Health Services; Veterinary Care and Food Safety; Administration, Control and General Affairs; and Programming and Integration of Health Services.

Each LHA's Department of Hygiene and Preventive Care governs an Infectious Disease Territorial Unit (IDTU) responsible for receiving infectious disease alerts from primary care services and hospitals in the district, alerting the territorial health authorities to potential new outbreaks (Ministry of Health Decree, 15 December 1990, "Sistema informativo delle malattie infettive e diffusive"), contact tracing and providing prophylaxis for the most common diseases. At the beginning of the pandemic, each IDTU was overwhelmed by the number of alert notifications and contact tracing required. Extra resources were organised to manage responses and speed up the contact tracing process, including: secondment of employees from other departments to each IDTU; integration of the laboratory-based surveillance system containing the PCR results of swab tests with the IDTU's symptoms-based surveillance system; development of bespoke software to easily allow GPs to flag patients suspected of being infected with SARS-CoV-2; and the creation of Special Units of medical personnel to supply residential care in each district.

For GPs in particular, from the 11th March 2020, precautionary measures were taken: in place of ambulatory practice, telephone counselling was instead offered to patients, with patients visiting GPs in their office only under specific request; and GPs were instructed to hand out important information on clinical indications, patient management and instructions for home isolation, if a patient was suspected to have COVID-19.

Following the increase in cases diagnosed in care homes, from the 30th March 2020 these were closed to visitors and care home-specific guidelines were handed out to healthcare workers to organise strict disease surveillance and better management of the cases in care homes (Welfare Directorate of Regione Lombardia 2020).

System	Name	Functions
National Health System	Italian Ministry of Health	The parliament sets the Essential Levels of Care (LEA) and the National Health Fund. Agreements are made between the IMH and the regional governments for the monitoring of LEA-related indicators, objectives for implementation and quality assurance, and budget criteria for fund allocation.
Regional Healthcare System	General Directorate of Welfare of Regione Lombardia	1) Defines budgets, rules, objectives for Local Health Authorities, which are the payers, and for hospital and clinic trusts (called Territorial Health Social Trusts), which are the providers;<br>2) Coordinates and supports Local Health Authorities;<br>3) Manages regional "monitoring system" (database)<br>4) Reports performance indicators to IMH.
	8 Local Health Authorities	1) Make agreements with the Territorial Health Social Trusts and with the hospitals with regards to the amount of medical services needed by the population.<br>2) Report data to General Directorate of Welfare of Regione Lombardia<br>3) Develop performance indicators and population-based indicators<br>4) Evaluate the performance of each hospital
	Public accredited hospitals and private accredited hospitals	1) Provide medical and surgical health services<br>2) Report data to LHA
	General Practitioners	1) Provide medical health services<br>2) Report data to LHA

Appendix Table 1. Regional Healthcare System of Regione Lombardia

Multi-state model structure

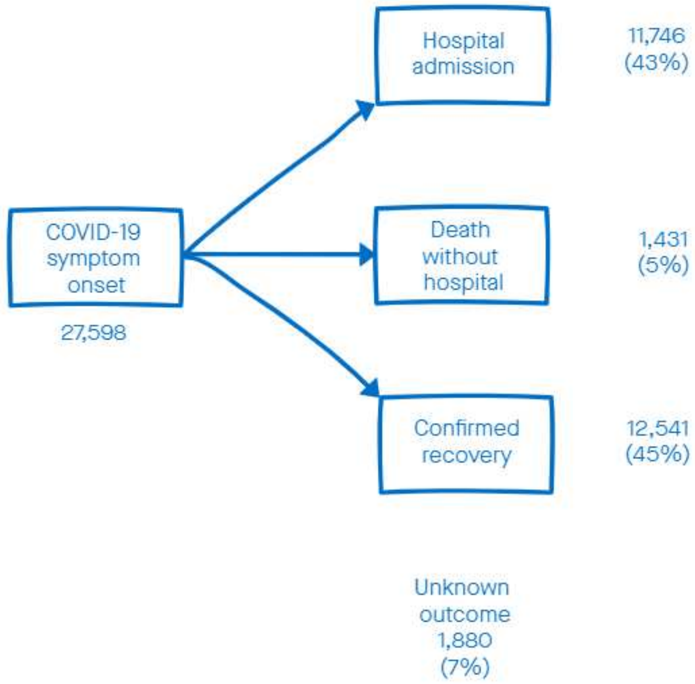

Appendix Figure 1. Multi-state model for next event after COVID-19 onset, and numbers of patients observed to experience each event.

Full results: probabilities of competing events

Summary of observed events by patient subgroup

	Admission	Death	Confirmed recovery	Unknown
Overall	11746 (43%)	1431 (5%)	12541 (45%)	1880 (7%)
Age 0-45	1623 (27%)	3 (0%)	3733 (63%)	552 (9%)
Age 45-65	3913 (46%)	28 (0%)	4193 (49%)	367 (4%)
Age 65+	6210 (47%)	1400 (11%)	4615 (35%)	961 (7%)
Female	4759 (31%)	949 (6%)	8439 (55%)	1160 (8%)
Male	6987 (57%)	482 (4%)	4102 (33%)	720 (6%)
Onset in February	1143 (72%)	20 (1%)	380 (24%)	45 (3%)
Onset in March	7971 (56%)	607 (4%)	5058 (35%)	661 (5%)
Onset in April	2126 (26%)	733 (9%)	4703 (57%)	724 (9%)
Onset in May	404 (16%)	71 (3%)	1859 (72%)	256 (10%)
Onset in June	102 (12%)	0 (0%)	541 (65%)	194 (23%)
No comorbidities	4854 (33%)	380 (3%)	8265 (56%)	1234 (8%)
Comorbidity	6892 (54%)	1051 (8%)	4276 (33%)	646 (5%)
Not care home resident	11070 (51%)	456 (2%)	8999 (41%)	1224 (6%)
Care home resident	676 (12%)	975 (17%)	3542 (61%)	656 (11%)
Not healthcare worker	11196 (46%)	1431 (6%)	9711 (40%)	1751 (7%)
Healthcare worker	550 (16%)	0 (0%)	2830 (81%)	129 (4%)

Appendix Table 2. Observed frequency of events following COVID-19 onset, for subgroups of interest.

Probabilities of death without hospital admission

Sex	Care home resident	At least one comorbidity	February	March	April	May	June
Female	No	No	2.5% (1.5, 3.9)	7.6% (6.2, 8.5)	7.7% (5.9, 8.9)	1.9% (1.1, 3.3)	2.2% (1.3, 4)
Female	Yes	No	13.5% (6.2, 24)	22.3% (19.5, 24.9)	14.5% (12.9, 16)	5.1% (4, 6.5)	4.9% (3.9, 6.2)
Female	No	Yes	2.2% (1.4, 3.6)	6.7% (5.8, 7.5)	8.3% (6.8, 9.9)	2.5% (1.6, 5.2)	1.3% (0.1, 4.4)
Female	Yes	Yes	15.1% (7.4, 25.9)	26.4% (24.5, 28.9)	18.2% (16.8, 19.9)	6.9% (5.6, 8.9)	4.8% (1, 7.7)
Male	No	No	2.1% (1.4, 3.3)	6.1% (5.1, 6.9)	7.6% (6.1, 8.9)	2.2% (1.2, 3.8)	2.9% (1.6, 5.6)
Male	Yes	No	15.1% (6.8, 25.6)	25.9% (22.8, 29.6)	18% (15.5, 20.1)	6.8% (5.2, 8.5)	6.7% (5.2, 8.5)
Male	No	Yes	1.8% (1.2, 2.9)	5.1% (4.6, 5.5)	7.4% (6.4, 8.7)	2.8% (1.6, 5.9)	1.7% (0.2, 5.8)
Male	Yes	Yes	15.9% (7.4, 27.1)	28.8% (26, 32.4)	21.6% (19.8, 23.3)	9% (7.4, 11.4)	6.4% (1.5, 10.1)

Appendix Table 3. Probabilities of death before hospital admission, after COVID-19 onset, for people over 65 years of age who were not healthcare workers, by patient characteristic and month of onset. Missing outcomes considered as censoring (as in main Figure 1).

Sex	Care home resident	At least one comorbidity	February	March	April	May	June
Female	No	No	2.3% (1.6, 4)	8% (6.7, 9.2)	8.7% (7, 11.1)	2.2% (1.4, 3.4)	2.4% (1.6, 3.5)
Female	Yes	No	13.7% (6.1, 24.8)	25.4% (22.9, 28.1)	16.6% (15.2, 18.7)	5.9% (4.2, 7.3)	5.6% (4.1, 6.8)
Female	No	Yes	2.2% (1.3, 3.6)	6.9% (6, 7.9)	9.1% (7.6, 11)	2.8% (1.7, 4.3)	1.5% (0.3, 4.4)
Female	Yes	Yes	15.5% (7.9, 26.1)	29.1% (26.8, 31.7)	20.4% (19.1, 22.1)	7.7% (5.8, 10)	5.4% (1.9, 8.6)
Male	No	No	2.1% (1.3, 3.4)	6.2% (5.4, 7.2)	8.1% (6.5, 10.3)	2.4% (1.5, 3.8)	3.2% (2, 4.9)
Male	Yes	No	15.4% (7, 28.3)	28.8% (25.8, 32.9)	20.2% (17.9, 23.1)	7.7% (5.5, 9.8)	7.6% (5.7, 9.7)
Male	No	Yes	1.9% (1.2, 3.3)	5.2% (4.6, 5.8)	7.9% (6.7, 9.8)	3% (1.8, 4.6)	1.9% (0.3, 6.2)
Male	Yes	Yes	16.6% (8.4, 29.4)	31.1% (27.6, 34.7)	23.6% (21.2, 25.5)	10.1% (7.6, 12.8)	7.3% (2.4, 12.2)

Appendix Table 4. Probabilities of death before hospital admission, after COVID-19 onset, for people over 65 years of age who were not healthcare workers, by patient characteristic and month of onset. Missing outcomes excluded.

Probabilities of hospital admission

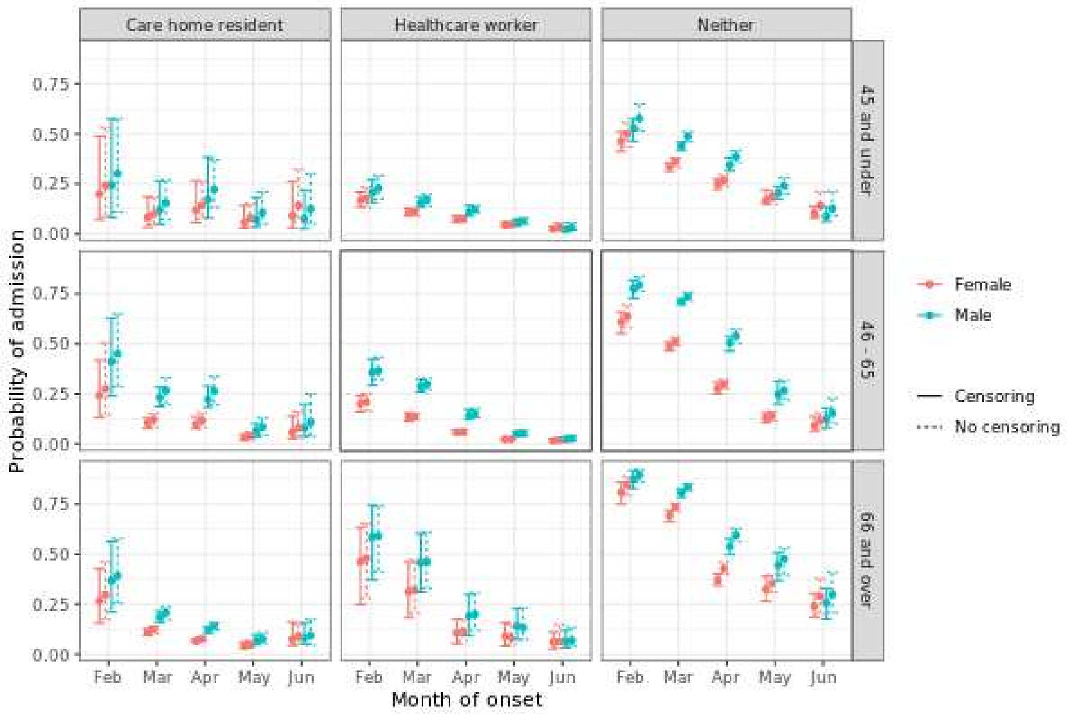

Appendix Figure 2. Probability of hospital admission following COVID-19 onset, for people without comorbidities, comparing by month of onset, age group, gender and whether a person is a care home resident, or a healthcare worker, or neither, for models with and without censored data included.

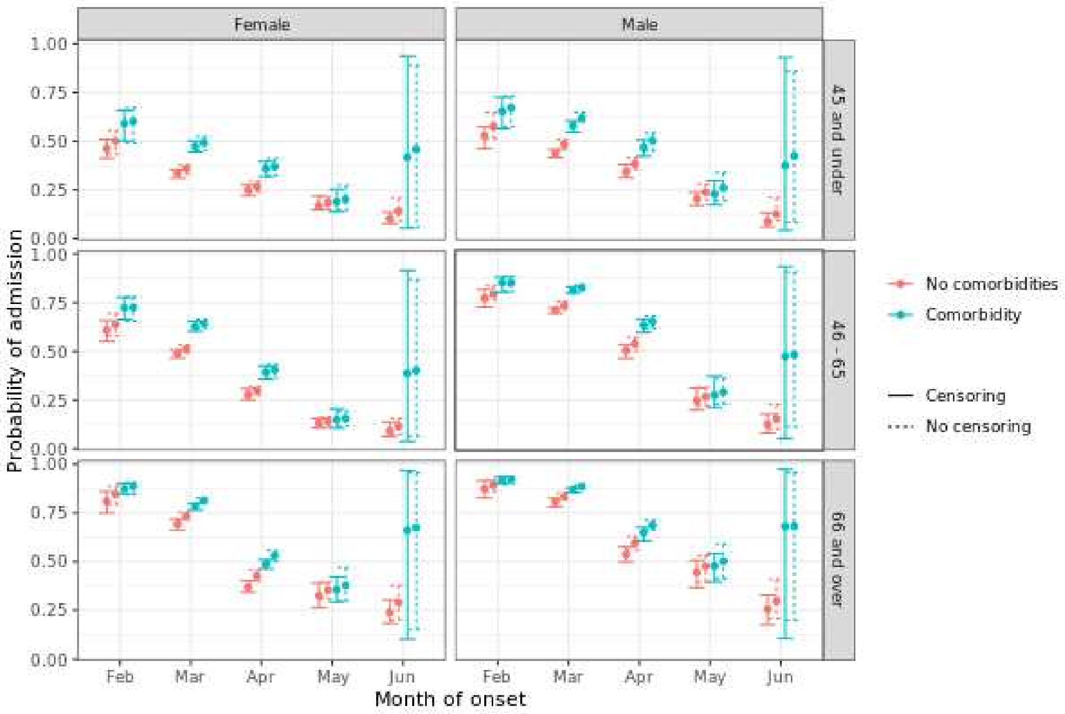

Appendix Figure 3. Probability of hospital admission following COVID-19 onset, for people who are not nursing home residents or healthcare workers, comparing people with and without comorbidities, by age group, gender and month of COVID-19 onset, for models with and without censored data included.

Age	Sex	Occupational status (healthcare worker, care home resident or neither)	One or more comorbidities	February	March	April	May	June
45 and under	Female	Neither	No comorbidities	46.2% (39.5, 52.2)	33.7% (31.8, 35.7)	24.9% (22.9, 27.5)	16.7% (14.1, 20.4)	10.3% (7.4, 14.3)
45 and under	Female	Care home resident	No comorbidities	19.9% (7.4, 47.9)	8.1% (4.8, 18.2)	11.7% (6.8, 25.6)	5.7% (2.8, 13.7)	8.9% (3.3, 19.9)
45 and under	Female	Healthcare worker	No comorbidities	16.7% (12.5, 21.5)	10.6% (9.2, 11.9)	7.2% (5.9, 9.1)	4.5% (3.6, 5.5)	2.6% (1.7, 3.9)
45 and under	Female	Neither	Comorbidity	59.1% (47.4, 65.6)	47.4% (45.1, 51.1)	36% (32.8, 39.5)	18.9% (14.4, 26)	41.8% (6.4, 93.2)
45 and under	Female	Care home resident	Comorbidity	28.2% (11.6, 57.1)	12.5% (7.4, 27.1)	17.6% (9.8, 35)	6.4% (3.4, 14.4)	37.7% (6.7, 91)
45 and under	Female	Healthcare worker	Comorbidity	25.2% (17.3, 33.1)	17.4% (15.7, 20.4)	11.6% (9.5, 14.2)	5.2% (3.7, 7.5)	14.4% (1.6, 75.7)
45 and under	Male	Neither	No comorbidities	52.8% (48, 59.1)	43.8% (40.7, 45.8)	34.2% (31.7, 36.7)	20.4% (16.7, 24.5)	8.7% (5.6, 13.7)
45 and under	Male	Care home resident	No comorbidities	24.3% (10, 51.9)	11.9% (6.8, 24.2)	17.1% (10.4, 33.5)	7.1% (3.4, 16.7)	7.6% (2.9, 17.7)
45 and under	Male	Healthcare worker	No comorbidities	20.7% (16.8, 25.3)	15.4% (13.4, 16.9)	10.8% (9.5, 13.1)	5.7% (4.2, 6.8)	2.2% (1.3, 3.8)
45 and under	Male	Neither	Comorbidity	65.3% (54.4, 70.4)	57.9% (55.4, 60.5)	46.9% (43, 49.6)	22.9% (18.1, 30.1)	37.5% (6.6, 92.4)
45 and under	Male	Care home resident	Comorbidity	33.1% (15.2, 60.7)	17.3% (10.3, 34.2)	24.6% (14.8, 43.2)	8% (4.8, 17.2)	33.5% (5.7, 89)
45 and under	Male	Healthcare worker	Comorbidity	30.5% (21.1, 36.7)	24.3% (21.2, 27.8)	17.1% (14.4, 20.2)	6.5% (4.4, 9)	12.3% (1.5, 72.9)
46 - 65	Female	Neither	No comorbidities	60.9% (55.2, 66.9)	48.7% (46.6, 50.7)	27.7% (25.9, 30.9)	13.2% (10.5, 16.1)	9.2% (6.3, 13.7)
46 - 65	Female	Care home resident	No comorbidities	24.2% (11.4, 41.1)	10.5% (7.3, 14.7)	9.8% (6.7, 13.7)	3.1% (1.7, 6)	5.8% (1.8, 11.8)
46 - 65	Female	Healthcare worker	No comorbidities	20.2% (16.6, 24.9)	13.3% (11.9, 14.4)	5.9% (5.1, 6.9)	2.4% (2, 3.1)	1.6% (1, 2.4)
46 - 65	Female	Neither	Comorbidity	72.4% (64.2, 76.6)	62.7% (61, 64.6)	39.4% (36.2, 43.3)	14.9% (11.3, 20.1)	38.8% (8.6, 91.6)
46 - 65	Female	Care home resident	Comorbidity	33.5% (16, 52.2)	15.9% (11.4, 21.2)	14.9% (10.9, 19.3)	3.5% (2, 6.8)	27.5% (4.7, 78.2)
46 - 65	Female	Healthcare worker	Comorbidity	29.8% (21.4, 34.6)	21.4% (19.4, 23.4)	9.5% (8, 11.2)	2.8% (2.1, 4)	9.3% (1.3, 64.2)
46 - 65	Male	Neither	No comorbidities	77.5% (74.5, 82)	71.2% (69.6, 72.8)	50.6% (48.4, 54.3)	24.8% (21.3, 30.3)	12.6% (8.4, 16.4)
46 - 65	Male	Care home resident	No comorbidities	41.2% (23.2, 60.5)	23.2% (16.6, 31)	22.3% (15.9, 29.3)	6.5% (3.7, 12.1)	8% (3.1, 15.2)

46 - 65	Male	Healthcare worker	No comorbidities	35.9% (31.6, 41.7)	28.7% (25.4, 31.9)	14.2% (12, 16.7)	5.1% (3.9, 6.4)	2.3% (1.4, 3.3)
46 - 65	Male	Neither	Comorbidity	85.3% (81.5, 87.8)	81.4% (80.2, 82.8)	63.4% (60.2, 67.1)	27.6% (22.9, 35.6)	47.4% (12.5, 93.8)
46 - 65	Male	Care home resident	Comorbidity	51.9% (30.7, 70.7)	32% (24.2, 40.8)	31.2% (23.5, 38.9)	7.3% (4.1, 13.2)	34.8% (7, 85.6)
46 - 65	Male	Healthcare worker	Comorbidity	48.5% (40.1, 52.8)	41.5% (37.6, 45.7)	22% (18.8, 25.2)	5.8% (4.5, 7.9)	12.8% (2, 71.1)
66 and over	Female	Neither	No comorbidities	80.9% (77.6, 86.4)	69.2% (66, 71.5)	36.9% (34.4, 39.4)	32.5% (27.8, 37.4)	23.9% (17.5, 32.3)
66 and over	Female	Care home resident	No comorbidities	26.6% (14, 42.4)	11% (9.6, 12.3)	6.6% (6.1, 7.5)	4.2% (2.9, 5.4)	7.5% (3.7, 12.6)
66 and over	Female	Healthcare worker	No comorbidities	46.2% (29.8, 60.6)	31.2% (19.7, 44.9)	10.6% (5.9, 16.8)	8.8% (5, 14.5)	6% (2.7, 10.8)
66 and over	Female	Neither	Comorbidity	87.2% (83.7, 89.9)	78.5% (77, 80.3)	48.8% (46, 50.5)	35.5% (29.6, 42.5)	66.1% (21.1, 97.1)
66 and over	Female	Care home resident	Comorbidity	36.3% (20.2, 53.4)	16.7% (14.9, 18.3)	10.2% (8.8, 11.2)	4.7% (3, 6.4)	33.3% (7, 86.8)
66 and over	Female	Healthcare worker	Comorbidity	58% (43.3, 67.6)	42.5% (28.2, 56.7)	16.1% (9.1, 25.1)	10% (5.6, 17.7)	28.3% (3.5, 85.5)
66 and over	Male	Neither	No comorbidities	87.4% (85.1, 90.9)	80.6% (79.2, 82.1)	53.7% (50.7, 57.4)	44.5% (36, 50.2)	25.6% (17.9, 33.3)
66 and over	Male	Care home resident	No comorbidities	36.8% (20.4, 54.3)	18.6% (16.6, 21)	12.1% (11.1, 13.7)	6.7% (4.4, 9)	8.1% (3.9, 15.6)
66 and over	Male	Healthcare worker	No comorbidities	58.4% (40.1, 72.7)	45.7% (32.1, 61.2)	19% (10.9, 29.3)	14% (6.7, 23)	6.5% (3.2, 13.1)
66 and over	Male	Neither	Comorbidity	91.7% (89.4, 93.7)	86.9% (86.1, 87.7)	65% (61.1, 66.9)	47.8% (42, 56.6)	68% (25.9, 97.4)
66 and over	Male	Care home resident	Comorbidity	47.4% (29.7, 63.9)	26.6% (24.2, 28.8)	17.9% (16.1, 19.5)	7.5% (5.1, 10.6)	35% (6.9, 85.9)
66 and over	Male	Healthcare worker	Comorbidity	69% (54.3, 79.2)	57.2% (41.9, 70.9)	27.3% (16, 41)	15.6% (8, 27.5)	30.1% (4.7, 87.4)

Appendix Table 5. Probability of hospital admission after COVID-19 onset, by patient characteristics and month of onset. Missing outcomes considered as censoring.

Age	Sex	Risk group (healthcare worker, care home resident or neither)	One or more comorbidities	February	March	April	May	June
45 and under	Female	Neither	No comorbidities	50% (43.2, 59.6)	36% (33.8, 38)	26.7% (23.4, 29)	18.4% (15.9, 20.5)	14.1% (10.7, 18.5)
45 and under	Female	Care home resident	No comorbidities	24.1% (9.7, 51.3)	10% (5.6, 19.4)	14.2% (7.5, 24.3)	7.8% (4, 16.2)	14.1% (5.4, 42.6)
45 and under	Female	Healthcare worker	No comorbidities	17.8% (13.2, 24.2)	10.9% (9.3, 12.3)	7.3% (6.1, 8.1)	4.7% (3.7, 5.8)	3.4% (2.3, 4.8)
45 and under	Female	Neither	Comorbidity	60.1% (48.6, 70.9)	49.3% (46.5, 52.6)	36.9% (32.6, 40.4)	20.3% (15, 26.5)	45.8% (4.2, 89)
45 and under	Female	Care home resident	Comorbidity	31% (13.5, 65.3)	14.7% (8.3, 27)	20% (10.9, 32)	8.5% (4.6, 17.2)	45.2% (5, 84.6)
45 and under	Female	Healthcare worker	Comorbidity	24.7% (17.7, 35.9)	17.4% (15, 19.6)	11.3% (9.4, 12.6)	5.2% (3.8, 7.5)	15.5% (1, 62.7)
45 and under	Male	Neither	No comorbidities	57.6% (50.8, 65.3)	48.5% (46.4, 50.3)	38.6% (35.6, 40.6)	23.9% (20.4, 27.8)	12.5% (8.3, 19.5)
45 and under	Male	Care home resident	No comorbidities	30% (13, 57.1)	15.4% (8.8, 27.6)	22.1% (12.7, 36.4)	10.5% (5.6, 21.3)	12.5% (4.3, 35.9)
45 and under	Male	Healthcare worker	No comorbidities	22.8% (17.2, 30.4)	17% (14.3, 18.6)	12% (10.1, 13.3)	6.4% (5.1, 8.1)	3% (1.9, 5.3)
45 and under	Male	Neither	Comorbidity	67.2% (56.1, 76.9)	61.9% (59.2, 64.2)	50.2% (45.7, 54.3)	26.1% (20.5, 35.3)	42.3% (3.1, 87.6)
45 and under	Male	Care home resident	Comorbidity	37.1% (16.8, 70.9)	21.5% (11.9, 37.8)	29.5% (17.7, 46.3)	11.4% (5.8, 21.9)	41.6% (3.1, 82.4)
45 and under	Male	Healthcare worker	Comorbidity	30.8% (22.4, 44.5)	26.1% (22.3, 28.9)	17.9% (14.9, 21.1)	7.1% (5, 10.2)	13.8% (0.7, 60.8)
46 - 65	Female	Neither	No comorbidities	63.7% (58.4, 68.8)	51.1% (49.3, 53.2)	29.9% (27.4, 32.1)	14.1% (11.8, 16.9)	11.6% (8, 16.9)
46 - 65	Female	Care home resident	No comorbidities	27.5% (14.1, 44.2)	12.3% (9.2, 17.4)	11.6% (9.1, 14.5)	4% (2.6, 6.1)	8.2% (3.6, 14.9)
46 - 65	Female	Healthcare worker	No comorbidities	21% (17.9, 24.8)	13.6% (12.3, 15.5)	6% (5, 6.9)	2.4% (2, 3.1)	1.9% (1.3, 3.1)
46 - 65	Female	Neither	Comorbidity	72.6% (67.7, 79.3)	64.3% (62.4, 66.7)	40.6% (37, 44)	15.6% (11.1, 19.8)	40.2% (3.9, 84.8)
46 - 65	Female	Care home resident	Comorbidity	35% (20.3, 57.8)	17.8% (13.9, 24.1)	16.6% (12.9, 20)	4.4% (2.7, 7.1)	30.9% (2.3, 73.6)
46 - 65	Female	Healthcare worker	Comorbidity	28.6% (24.3, 37.5)	21.4% (19.8, 23.8)	9.3% (7.9, 10.7)	2.7% (1.8, 3.8)	9.2% (0.6, 44.8)
46 - 65	Male	Neither	No comorbidities	79.3% (76, 83.4)	73.7% (72.5, 74.9)	53.9% (51, 57.7)	26.8% (23.2, 31.6)	15.4% (9.6, 21.7)

46 - 65	Male	Care home resident	No comorbidities	45% (26, 62.5)	26.9% (20.6, 34.7)	26.4% (20.5, 30.9)	8.5% (5.6, 12)	11% (4.9, 20.9)
46 - 65	Male	Healthcare worker	No comorbidities	36.6% (32.5, 42.6)	29.7% (26.7, 32.5)	15% (12.5, 17)	5.2% (4.3, 6.6)	2.7% (1.6, 4)
46 - 65	Male	Neither	Comorbidity	85.2% (81.6, 88.8)	82.8% (81.7, 83.9)	65.2% (62.5, 68.8)	29.1% (23.1, 36.6)	48.3% (4.2, 88.5)
46 - 65	Male	Care home resident	Comorbidity	53.1% (33.4, 72.4)	35.5% (28.3, 43.6)	34.7% (27.3, 41.6)	9.2% (5.2, 13.9)	38.1% (2.2, 81.6)
46 - 65	Male	Healthcare worker	Comorbidity	46.5% (40.3, 55.3)	42.1% (39.7, 44.6)	22.1% (18.8, 25.3)	5.8% (4, 8.4)	12.4% (0.7, 53.2)
66 and over	Female	Neither	No comorbidities	80.9% (77.6, 86.4)	69.2% (66, 71.5)	36.9% (34.4, 39.4)	32.5% (27.8, 37.4)	23.9% (17.5, 32.3)
66 and over	Female	Care home resident	No comorbidities	26.6% (14, 42.4)	11% (9.6, 12.3)	6.6% (6.1, 7.5)	4.2% (2.9, 5.4)	7.5% (3.7, 12.6)
66 and over	Female	Healthcare worker	No comorbidities	46.2% (29.8, 60.6)	31.2% (19.7, 44.9)	10.6% (5.9, 16.8)	8.8% (5, 14.5)	6% (2.7, 10.8)
66 and over	Female	Neither	Comorbidity	87.2% (83.7, 89.9)	78.5% (77, 80.3)	48.8% (46, 50.5)	35.5% (29.6, 42.5)	66.1% (21.1, 97.1)
66 and over	Female	Care home resident	Comorbidity	36.3% (20.2, 53.4)	16.7% (14.9, 18.3)	10.2% (8.8, 11.2)	4.7% (3, 6.4)	33.3% (7, 86.8)
66 and over	Female	Healthcare worker	Comorbidity	58% (43.3, 67.6)	42.5% (28.2, 56.7)	16.1% (9.1, 25.1)	10% (5.6, 17.7)	28.3% (3.5, 85.5)
66 and over	Male	Neither	No comorbidities	87.4% (85.1, 90.9)	80.6% (79.2, 82.1)	53.7% (50.7, 57.4)	44.5% (36, 50.2)	25.6% (17.9, 33.3)
66 and over	Male	Care home resident	No comorbidities	36.8% (20.4, 54.3)	18.6% (16.6, 21)	12.1% (11.1, 13.7)	6.7% (4.4, 9)	8.1% (3.9, 15.6)
66 and over	Male	Healthcare worker	No comorbidities	58.4% (40.1, 72.7)	45.7% (32.1, 61.2)	19% (10.9, 29.3)	14% (6.7, 23)	6.5% (3.2, 13.1)
66 and over	Male	Neither	Comorbidity	91.7% (89.4, 93.7)	86.9% (86.1, 87.7)	65% (61.1, 66.9)	47.8% (42, 56.6)	68% (25.9, 97.4)
66 and over	Male	Care home resident	Comorbidity	47.4% (29.7, 63.9)	26.6% (24.2, 28.8)	17.9% (16.1, 19.5)	7.5% (5.1, 10.6)	35% (6.9, 85.9)
66 and over	Male	Healthcare worker	Comorbidity	69% (54.3, 79.2)	57.2% (41.9, 70.9)	27.3% (16, 41)	15.6% (8, 27.5)	30.1% (4.7, 87.4)

Appendix Table 6. Probability of hospital admission after COVID-19 onset, by patient characteristics and month of onset. Missing outcomes excluded.

Full results: times to events

Summary of times to observed events

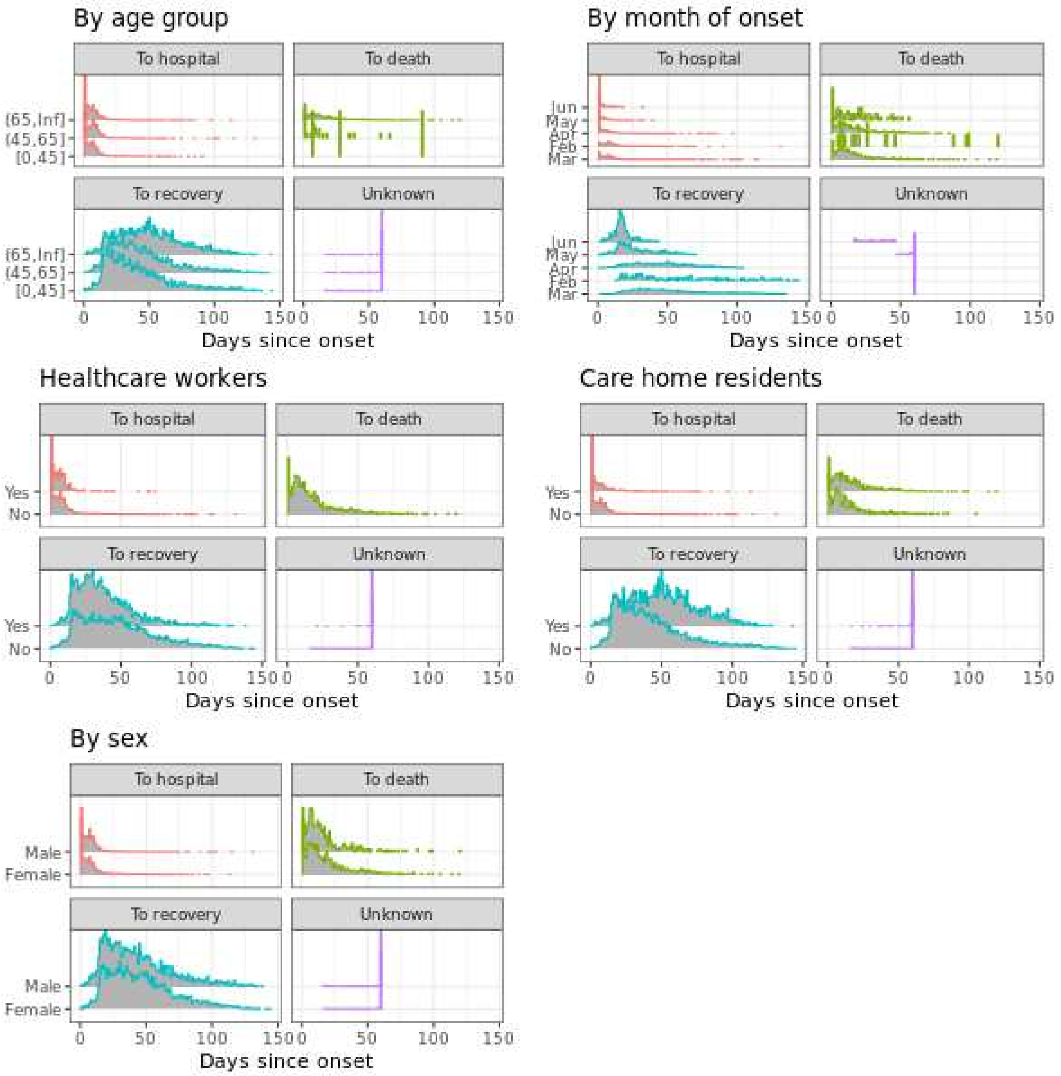

Appendix Figure 4. Histograms showing the frequencies (by subgroup) of observed times from COVID-19 onset to alternative events (hospital admission, death without admission or recovery without admission), or time to censoring for those whose next event was unknown.

Appendix Figure 4 illustrates the distribution of the observed times from onset to hospital admission, death without admission and confirmed recovery without admission, and times to the assumed date of censoring (minimum of time to data extraction and 60 days) for people who had none of these events recorded.

Combining all individuals and neglecting censoring, the median time from onset to admission was 6 days (interquartile range 2 to 10 days), the median time to death without admission was 12 days (interquartile range 6 to 22 days) and the median time to confirmed recovery was 41 days (interquartile range 26 to 60 days). Recall that this is defined as the time to the

second negative test with no symptoms, therefore will be greater than the time to clinical recovery.

Summaries of times to events from the fitted models

Histograms of the observed times to events are shown in Appendix Figure 4. From the fitted model, times to admission (Appendix Figure 5) become shorter from February to June (from median 7 days to 1 day for males aged 66 and over who were not care home residents). Slightly longer times to admission were observed for care home residents (from median 10 days in February to 2 days in June). No substantial differences in times to admission were observed between people of different ages and genders, for healthcare workers or for people with comorbidities (see Appendix Figures 5-7 and Tables 7-12). Variability in times to admission became smaller from February (main manuscript Figure 3, 95% quantile interval 1-50 days for women and men over 65) to June (0-10 days)

For times to death without hospital admission, no significant covariate effects were observed, other than slightly higher times to death for February onsets (median 22 days (13 to 35) for men and 24 days (15 to 39) for women over 65 in the baseline risk group, compared to 10 days or less in later months). Variability in these times was highest in February (2-123 days for women over 65), but constant in subsequent months (1-54 days).

Shorter times to confirmed recovery (Appendix Figure 7) were estimated as time passed from February to June (median 74 to 19 for people aged 66 and over who were not healthcare workers or care home residents). Shorter times to confirmed recovery were also estimated for people under 65 (median 57 days in February). Variability between patients in time to recovery decreased from February (95% quantile interval 22-175 days for women over 65) to May (6-46 days).

The finding that younger groups appear to recover slightly faster than older groups is in accordance with the literature (Voinsky, Baristaite, and Gurwitz 2020; Castillo et al., 2020), with the difference possibly explained by the progressive decay of the immune system with age. The estimated time to PCR-confirmed recovery for those not hospitalised progressively decreases with calendar time. This may be explained by increasing testing capacity, either due to recovery being confirmed more rapidly, or because of changes in case-mix due to more cases of lower severity being identified and treated outside hospital following testing. Alternatively it may reflect improved capacity for treatment outside hospital as the first wave waned.

Times to events compared between subgroups.

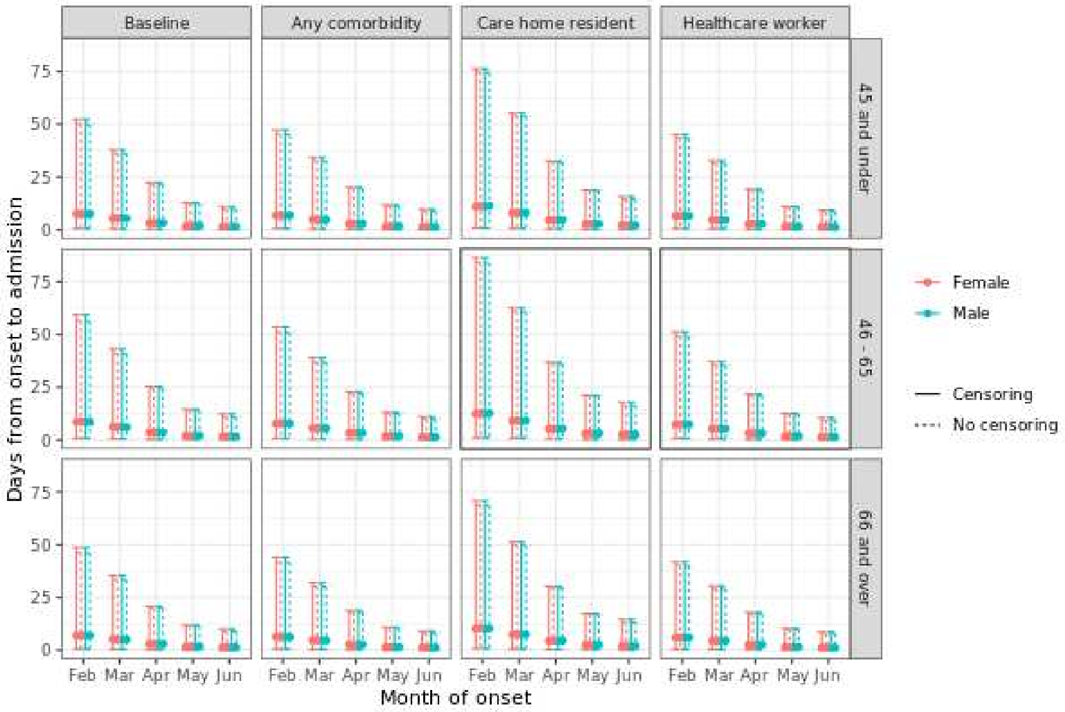

Appendix Figure 5. Times from COVID-19 onset to hospital admission (median and range containing 95% of individuals), by month of onset, age and gender, and comparing a baseline group with none of the following risk factors: healthcare workers, care home residents and people with comorbidities, to a group with one of these risk factors.

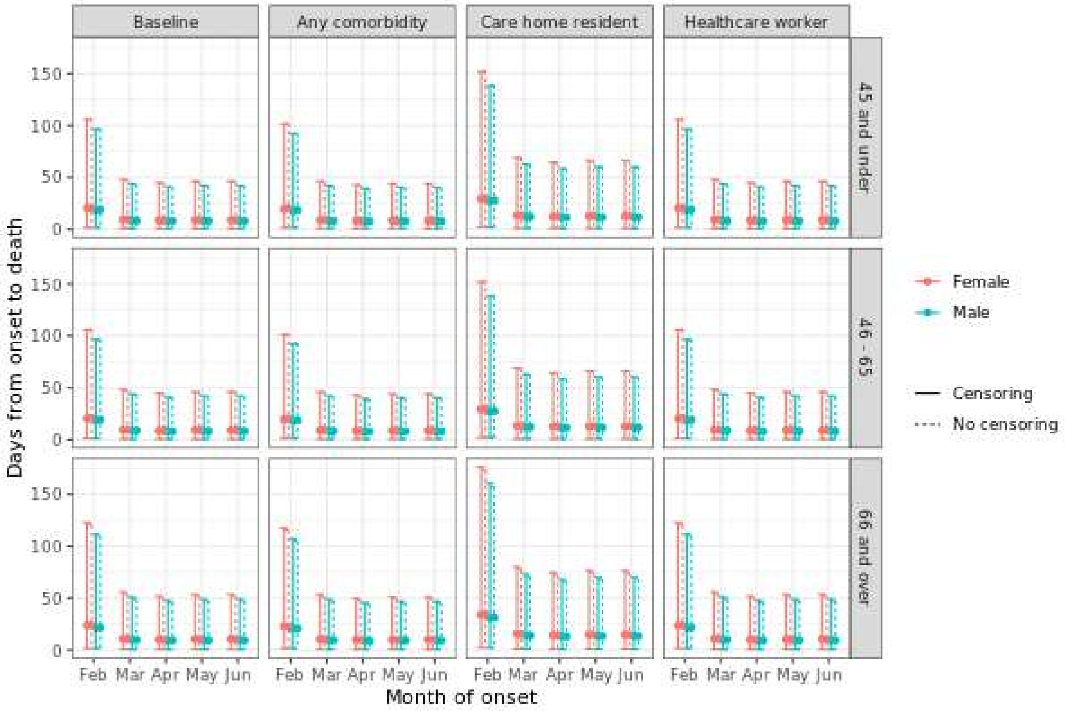

Appendix Figure 6. Times from COVID-19 onset to death without hospital admission (median and range containing 95% of individuals), by month of onset, age and gender, and comparing a baseline group with none of the following risk factors: healthcare workers, care home residents and people with comorbidities, to a group with one of these risk factors.

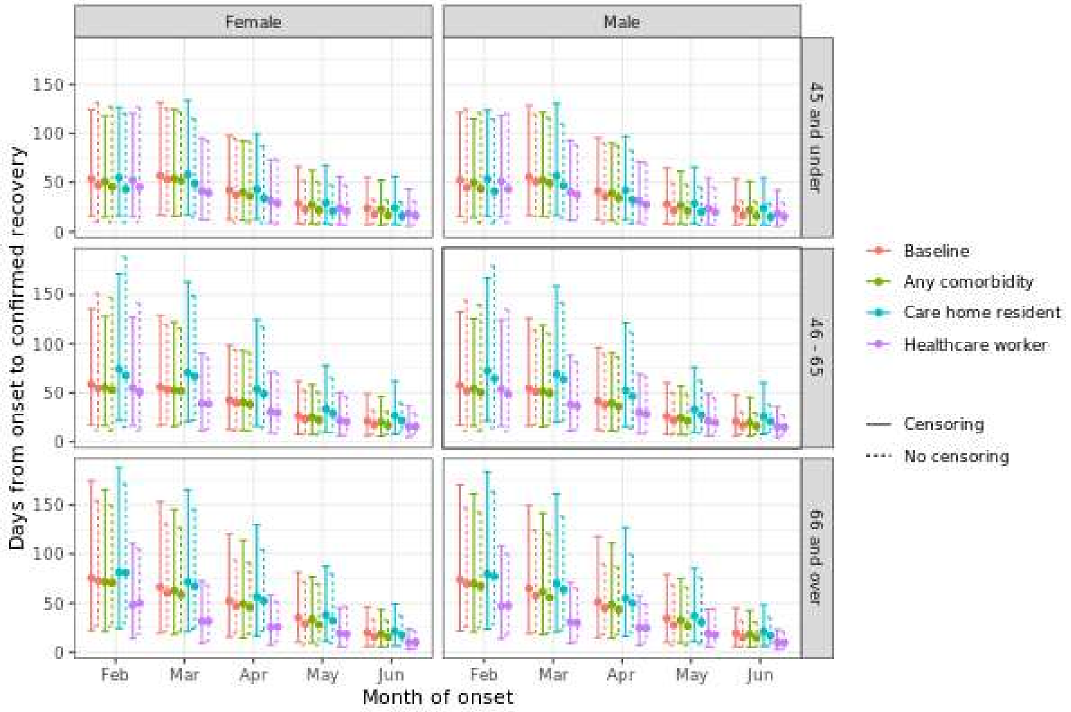

Appendix Figure 7. Times from COVID-19 onset to confirmed recovery (second negative test and no symptoms) (median and range containing 95% of individuals), by month of onset, age and gender, and comparing a baseline group with none of the following risk factors: healthcare workers, care home residents and people with comorbidities, to a group with one of these risk factors.

Appendix Figure 7 shows estimates of times to recovery by subgroups additional to those presented in the main text. Shorter times to confirmed recovery were estimated for people under 65 (median 57 days in February) and for healthcare workers (median 54 days in February to 16 days in June, considering those under 65), and slightly longer times to confirmed recovery for nursing home residents (median 80 days in February to 21 days in June, considering those over 65).

Times to events following COVID-19 onset (missing outcomes considered as censoring)

The following three tables present estimates of the times to the three potential alternative events following COVID-19 onset (hospital admission, death or recovery without admission) corresponding to the figures in the main manuscript, from a model where missing outcomes are considered as censoring.

			**February**	**March**	**April**	**May**	**June**
45 and under	Female	Baseline	7.5 (6.9-8) [0.6-52.4]	5.5 (5.1-5.8) [0.5-38]	3.2 (3-3.4) [0.3-22.4]	1.9 (1.6-2.1) [0.2-12.9]	1.6 (1.3-1.9) [0.1-10.8]
46 - 65	Female	Baseline	8.5 (7.9-9.2) [0.7-59.3]	6.2 (5.9-6.5) [0.5-43.1]	3.6 (3.4-3.9) [0.3-25.3]	2.1 (1.9-2.3) [0.2-14.6]	1.8 (1.4-2.2) [0.1-12.3]
66 and over	Female	Baseline	7 (6.4-7.5) [0.6-48.6]	5.1 (4.8-5.4) [0.4-35.3]	3 (2.8-3.2) [0.2-20.8]	1.7 (1.5-1.9) [0.1-11.9]	1.4 (1.2-1.8) [0.1-10.1]
45 and under	Male	Baseline	7.5 (6.9-8.1) [0.6-52.3]	5.5 (5.1-5.8) [0.5-38]	3.2 (3-3.5) [0.3-22.3]	1.9 (1.6-2.1) [0.2-12.9]	1.6 (1.3-1.9) [0.1-10.8]
46 - 65	Male	Baseline	8.5 (8-9.2) [0.7-59.3]	6.2 (5.9-6.5) [0.5-43]	3.6 (3.4-3.9) [0.3-25.3]	2.1 (1.9-2.3) [0.2-14.6]	1.8 (1.4-2.2) [0.1-12.3]
66 and over	Male	Baseline	7 (6.5-7.4) [0.6-48.6]	5.1 (4.8-5.4) [0.4-35.3]	3 (2.8-3.2) [0.2-20.7]	1.7 (1.6-1.9) [0.1-11.9]	1.4 (1.2-1.8) [0.1-10.1]
45 and under	Female	Care home resident	11 (9.6-12.2) [0.9-76.3]	8 (7.1-8.8) [0.7-55.4]	4.7 (4.1-5.2) [0.4-32.6]	2.7 (2.3-3.1) [0.2-18.7]	2.3 (1.8-2.8) [0.2-15.8]
46 - 65	Female	Care home resident	12.4 (11.1-13.7) [1-86.4]	9 (8.2-9.7) [0.8-62.7]	5.3 (4.8-5.8) [0.4-36.9]	3.1 (2.7-3.5) [0.3-21.2]	2.6 (2-3.2) [0.2-17.9]
66 and over	Female	Care home resident	10.2 (9-11.1) [0.9-70.8]	7.4 (6.8-8) [0.6-51.4]	4.3 (3.9-4.8) [0.4-30.2]	2.5 (2.2-2.9) [0.2-17.4]	2.1 (1.7-2.6) [0.2-14.7]
45 and under	Male	Care home resident	11 (9.6-12.2) [0.9-76.2]	8 (7-8.9) [0.7-55.3]	4.7 (4.1-5.2) [0.4-32.5]	2.7 (2.3-3.1) [0.2-18.7]	2.3 (1.8-2.9) [0.2-15.8]
46 - 65	Male	Care home resident	12.4 (11.1-13.9) [1-86.3]	9 (8.2-10) [0.8-62.6]	5.3 (4.7-5.9) [0.4-36.8]	3.1 (2.7-3.5) [0.3-21.2]	2.6 (2-3.2) [0.2-17.9]
66 and over	Male	Care home resident	10.2 (9.1-11.3) [0.8-70.7]	7.4 (6.6-8.2) [0.6-51.3]	4.3 (3.9-4.8) [0.4-30.2]	2.5 (2.2-2.9) [0.2-17.4]	2.1 (1.7-2.6) [0.2-14.6]
45 and under	Female	Healthcare worker	6.5 (5.8-7.2) [0.5-45.2]	4.7 (4.3-5.3) [0.4-32.8]	2.8 (2.5-3.1) [0.2-19.3]	1.6 (1.4-1.8) [0.1-11.1]	1.3 (1.1-1.7) [0.1-9.4]
46 - 65	Female	Healthcare worker	7.4 (6.6-8) [0.6-51.2]	5.3 (4.9-5.8) [0.4-37.2]	3.1 (2.8-3.5) [0.3-21.8]	1.8 (1.6-2) [0.2-12.6]	1.5 (1.2-1.9) [0.1-10.6]
66 and over	Female	Healthcare worker	6 (5.4-6.6) [0.5-42]	4.4 (4-4.8) [0.4-30.5]	2.6 (2.3-2.8) [0.2-17.9]	1.5 (1.3-1.7) [0.1-10.3]	1.3 (1-1.6) [0.1-8.7]

45 and under	Male	Healthcare worker	6.5 (5.9-7.2) [0.5-45.2]	4.7 (4.3-5.2) [0.4-32.8]	2.8 (2.5-3.1) [0.2-19.3]	1.6 (1.4-1.8) [0.1-11.1]	1.3 (1.1-1.6) [0.1-9.4]
46 - 65	Male	Healthcare worker	7.4 (6.7-8.1) [0.6-51.1]	5.3 (4.9-5.8) [0.4-37.1]	3.1 (2.9-3.4) [0.3-21.8]	1.8 (1.6-2.1) [0.2-12.6]	1.5 (1.2-1.9) [0.1-10.6]
66 and over	Male	Healthcare worker	6 (5.4-6.6) [0.5-41.9]	4.4 (4-4.8) [0.4-30.4]	2.6 (2.3-2.9) [0.2-17.9]	1.5 (1.3-1.7) [0.1-10.3]	1.2 (1-1.6) [0.1-8.7]
45 and under	Female	Any comorbidity	6.8 (6.2-7.4) [0.6-47.3]	4.9 (4.6-5.3) [0.4-34.4]	2.9 (2.7-3.2) [0.2-20.2]	1.7 (1.5-1.9) [0.1-11.6]	1.4 (1.1-1.8) [0.1-9.8]
46 - 65	Female	Any comorbidity	7.7 (7.2-8.3) [0.6-53.6]	5.6 (5.3-5.8) [0.5-38.9]	3.3 (3.1-3.5) [0.3-22.9]	1.9 (1.7-2.1) [0.2-13.2]	1.6 (1.3-2) [0.1-11.1]
66 and over	Female	Any comorbidity	6.3 (5.9-6.8) [0.5-43.9]	4.6 (4.4-4.8) [0.4-31.9]	2.7 (2.5-2.8) [0.2-18.8]	1.6 (1.4-1.7) [0.1-10.8]	1.3 (1.1-1.6) [0.1-9.1]
45 and under	Male	Any comorbidity	6.8 (6.2-7.5) [0.6-47.3]	4.9 (4.6-5.4) [0.4-34.3]	2.9 (2.7-3.2) [0.2-20.2]	1.7 (1.5-1.9) [0.1-11.6]	1.4 (1.1-1.8) [0.1-9.8]
46 - 65	Male	Any comorbidity	7.7 (7.2-8.2) [0.6-53.6]	5.6 (5.4-5.8) [0.5-38.9]	3.3 (3.1-3.5) [0.3-22.9]	1.9 (1.7-2.1) [0.2-13.2]	1.6 (1.3-2) [0.1-11.1]
66 and over	Male	Any comorbidity	6.3 (6-6.7) [0.5-43.9]	4.6 (4.5-4.7) [0.4-31.9]	2.7 (2.5-2.9) [0.2-18.7]	1.6 (1.4-1.8) [0.1-10.8]	1.3 (1.1-1.6) [0.1-9.1]

Appendix Table 7. Days to hospital admission after COVID-19 onset, as in Figure 5, by month of onset, age, sex and risk group (health care workers, care home residents, people with comorbidities, and a baseline with none of these risk factors). Times shown in days, in the following form: median (95% confidence interval for median) [95% quantiles representing variability between individuals]

Age	Sex	Risk group	February	March	April	May	June
45 and under	Female	Baseline	20.4 (11.4-35.3) [1.4-105.7]	9.3 (6.8-13) [0.7-48.1]	8.7 (6.1-12) [0.6-44.8]	8.9 (5.8-13.1) [0.6-46.1]	8.9 (5.8-13.1) [0.6-46.1]
45 and under	Female	Care home resident	29.3 (17.4-51.2) [2.1-151.6]	13.4 (9.4-18.1) [0.9-69.1]	12.4 (9.1-16.5) [0.9-64.2]	12.8 (8.6-18.7) [0.9-66.1]	12.8 (8.6-18.7) [0.9-66.1]
45 and under	Female	Healthcare worker	20.4 (11.4-35.3) [1.4-105.7]	9.3 (6.8-13) [0.7-48.1]	8.7 (6.1-12) [0.6-44.8]	8.9 (5.8-13.1) [0.6-46.1]	8.9 (5.8-13.1) [0.6-46.1]
45 and under	Female	Any comorbidity	19.6 (10.9-34.3) [1.4-101.1]	8.9 (6.2-12.1) [0.6-46.1]	8.3 (5.7-11) [0.6-42.9]	8.5 (5.4-12.8) [0.6-44.1]	8.5 (5.4-12.8) [0.6-44.1]
45 and under	Male	Baseline	18.6 (10.2-31.1) [1.3-96.4]	8.5 (6.2-11.7) [0.6-43.9]	7.9 (5.7-10.5) [0.6-40.8]	8.1 (5.3-11.9) [0.6-42]	8.1 (5.3-11.9) [0.6-42]
45 and under	Male	Care home resident	26.7 (15.5-44.9) [1.9-138.3]	12.2 (9-16.7) [0.9-63]	11.3 (8.4-15.3) [0.8-58.6]	11.7 (7.9-17.4) [0.8-60.3]	11.7 (7.9-17.4) [0.8-60.3]
45 and under	Male	Healthcare worker	18.6 (10.2-31.1) [1.3-96.4]	8.5 (6.2-11.7) [0.6-43.9]	7.9 (5.7-10.5) [0.6-40.8]	8.1 (5.3-11.9) [0.6-42]	8.1 (5.3-11.9) [0.6-42]
45 and under	Male	Any comorbidity	17.8 (10.2-30.5) [1.3-92.2]	8.1 (5.8-11.1) [0.6-42]	7.6 (5.2-10.1) [0.5-39.1]	7.8 (4.9-12.1) [0.6-40.2]	7.8 (4.9-12.1) [0.6-40.2]
46 - 65	Female	Baseline	20.4 (11.4-35.3) [1.4-105.7]	9.3 (6.8-13) [0.7-48.1]	8.7 (6.1-12) [0.6-44.8]	8.9 (5.8-13.1) [0.6-46.1]	8.9 (5.8-13.1) [0.6-46.1]
46 - 65	Female	Care home resident	29.3 (17.4-51.2) [2.1-151.6]	13.4 (9.4-18.1) [0.9-69.1]	12.4 (9.1-16.5) [0.9-64.2]	12.8 (8.6-18.7) [0.9-66.1]	12.8 (8.6-18.7) [0.9-66.1]
46 - 65	Female	Healthcare worker	20.4 (11.4-35.3) [1.4-105.7]	9.3 (6.8-13) [0.7-48.1]	8.7 (6.1-12) [0.6-44.8]	8.9 (5.8-13.1) [0.6-46.1]	8.9 (5.8-13.1) [0.6-46.1]
46 - 65	Female	Any comorbidity	19.6 (10.9-34.3) [1.4-101.1]	8.9 (6.2-12.1) [0.6-46.1]	8.3 (5.7-11) [0.6-42.9]	8.5 (5.4-12.8) [0.6-44.1]	8.5 (5.4-12.8) [0.6-44.1]
46 - 65	Male	Baseline	18.6 (10.2-31.1) [1.3-96.4]	8.5 (6.2-11.7) [0.6-43.9]	7.9 (5.7-10.5) [0.6-40.8]	8.1 (5.3-11.9) [0.6-42]	8.1 (5.3-11.9) [0.6-42]
46 - 65	Male	Care home resident	26.7 (15.5-44.9) [1.9-138.3]	12.2 (9-16.7) [0.9-63]	11.3 (8.4-15.3) [0.8-58.6]	11.7 (7.9-17.4) [0.8-60.3]	11.7 (7.9-17.4) [0.8-60.3]
46 - 65	Male	Healthcare worker	18.6 (10.2-31.1) [1.3-96.4]	8.5 (6.2-11.7) [0.6-43.9]	7.9 (5.7-10.5) [0.6-40.8]	8.1 (5.3-11.9) [0.6-42]	8.1 (5.3-11.9) [0.6-42]
46 - 65	Male	Any comorbidity	17.8 (10.2-30.5) [1.3-92.2]	8.1 (5.8-11.1) [0.6-42]	7.6 (5.2-10.1) [0.5-39.1]	7.8 (4.9-12.1) [0.6-40.2]	7.8 (4.9-12.1) [0.6-40.2]
66 and over	Female	Baseline	23.7 (14.6-38.6) [1.7-122.7]	10.8 (9.1-12.7) [0.8-55.9]	10.1 (8.6-11.8) [0.7-52]	10.4 (8-13.3) [0.7-53.5]	10.4 (8-13.3) [0.7-53.5]

66 and over	Female	Care home resident	34.1 (21.2-56.5) [2.4-176.1]	15.5 (13.5-18.2) [1.1-80.2]	14.4 (13-16.4) [1-74.6]	14.9 (12.1-19) [1.1-76.8]	14.9 (12.1-19) [1.1-76.8]
66 and over	Female	Healthcare worker	23.7 (14.6-38.6) [1.7-122.7]	10.8 (9.1-12.7) [0.8-55.9]	10.1 (8.6-11.8) [0.7-52]	10.4 (8-13.3) [0.7-53.5]	10.4 (8-13.3) [0.7-53.5]
66 and over	Female	Any comorbidity	22.7 (14-36.6) [1.6-117.5]	10.3 (9-11.6) [0.7-53.5]	9.6 (8.2-10.8) [0.7-49.8]	9.9 (7.7-12.5) [0.7-51.2]	9.9 (7.7-12.5) [0.7-51.2]
66 and over	Male	Baseline	21.6 (13.2-35.1) [1.5-111.9]	9.9 (8.4-11.9) [0.7-51]	9.2 (7.7-10.8) [0.6-47.4]	9.4 (7.2-12.5) [0.7-48.8]	9.4 (7.2-12.5) [0.7-48.8]
66 and over	Male	Care home resident	31.1 (20.2-50.4) [2.2-160.6]	14.1 (12.5-16.8) [1-73.2]	13.2 (11.9-15.2) [0.9-68.1]	13.6 (10.8-18) [1-70.1]	13.6 (10.8-18) [1-70.1]
66 and over	Male	Healthcare worker	21.6 (13.2-35.1) [1.5-111.9]	9.9 (8.4-11.9) [0.7-51]	9.2 (7.7-10.8) [0.6-47.4]	9.4 (7.2-12.5) [0.7-48.8]	9.4 (7.2-12.5) [0.7-48.8]
66 and over	Male	Any comorbidity	20.7 (12.7-32.8) [1.5-107.1]	9.4 (8.5-10.6) [0.7-48.8]	8.8 (7.6-10) [0.6-45.4]	9 (7.2-11.8) [0.6-46.7]	9 (7.2-11.8) [0.6-46.7]

Appendix Table 8. Days to death before hospital admission, after COVID-19 onset, as in Figure 6 , by month of onset, age, sex and risk group (health care workers, care home residents, people with comorbidities, and a baseline with none of these risk factors). Times shown in days, in the following form: median (95% confidence interval for median) [95% quantiles representing variability between individuals]

Age	Sex	Risk group	February	March	April	May	June
45 and under	Female	Baseline	53.8 (50-59.8) [15.9-124.1]	57 (56.6-57.4) [16.8-131.4]	42.3 (41.2-43.8) [12.5-97.6]	28.7 (27.6-29.7) [8.5-66.2]	23.9 (22.6-25.1) [7.1-55.1]
45 and under	Female	Care home resident	54.7 (47.4-63.8) [16.2-126.2]	58 (51.8-63.9) [17.1-133.6]	43 (38-47.7) [12.7-99.2]	29.2 (25.8-32.2) [8.6-67.3]	24.3 (21.4-27.3) [7.2-56]
45 and under	Female	Healthcare worker	52.4 (46.9-58.4) [15.5-120.7]	41.2 (40.2-42.4) [12.2-95.1]	31.5 (30.1-33.2) [9.3-72.7]	24.2 (22.3-26.3) [7.2-55.9]	18.6 (16.1-22.6) [5.5-43]
45 and under	Female	Any comorbidity	51 (47.3-56.2) [15.1-117.5]	54 (52.8-55.1) [15.9-124.4]	40.1 (38.7-41.5) [11.8-92.4]	27.2 (25.9-28.2) [8-62.7]	22.6 (21.3-23.9) [6.7-52.2]
45 and under	Male	Baseline	52.6 (49-58.4) [15.5-121.2]	55.7 (54.8-56.7) [16.4-128.4]	41.3 (40.1-42.9) [12.2-95.3]	28 (26.9-29) [8.3-64.6]	23.3 (22.1-24.6) [6.9-53.8]
45 and under	Male	Care home resident	53.5 (46.7-62.6) [15.8-123.3]	56.6 (50.5-62.7) [16.7-130.5]	42 (36.8-46.7) [12.4-96.9]	28.5 (24.9-31.3) [8.4-65.7]	23.7 (20.7-26.7) [7-54.7]
45 and under	Male	Healthcare worker	51.1 (46-57.2) [15.1-117.9]	40.3 (39.1-41.4) [11.9-92.9]	30.8 (29.3-32.3) [9.1-71]	23.7 (21.9-25.9) [7-54.6]	18.2 (15.6-22.2) [5.4-42]
45 and under	Male	Any comorbidity	49.8 (46.2-54.9) [14.7-114.8]	52.7 (51.5-54.2) [15.6-121.5]	39.1 (37.8-40.7) [11.6-90.2]	26.5 (25.3-27.4) [7.8-61.2]	22.1 (20.8-23.4) [6.5-51]
46 - 65	Female	Baseline	58.7 (54.6-63.3) [17.3-135.4]	55.9 (54.7-57.8) [16.5-128.8]	42.7 (41.4-44) [12.6-98.4]	26.7 (25.8-27.8) [7.9-61.6]	21.2 (19.5-22.9) [6.3-48.8]
46 - 65	Female	Care home resident	74.2 (66.1-82) [21.9-171.1]	70.6 (66.7-75.4) [20.9-162.8]	53.9 (50.4-57.1) [15.9-124.4]	33.8 (31.6-36.2) [10-77.9]	26.8 (24.6-30.2) [7.9-61.7]
46 - 65	Female	Healthcare worker	55.1 (50.7-60.6) [16.3-127.1]	39 (38.2-40.1) [11.5-90]	30.7 (29.4-32.2) [9.1-70.8]	21.8 (20.2-23.5) [6.4-50.2]	15.9 (13.7-19.4) [4.7-36.8]
46 - 65	Female	Any comorbidity	55.6 (50.9-60) [16.4-128.2]	52.9 (51.8-54.5) [15.6-122]	40.4 (39.3-41.9) [11.9-93.2]	25.3 (24.3-26.4) [7.5-58.3]	20.1 (18.2-21.9) [5.9-46.2]
46 - 65	Male	Baseline	57.4 (53.2-61.7) [16.9-132.2]	54.6 (53.4-56.3) [16.1-125.8]	41.7 (40.3-43.2) [12.3-96.1]	26.1 (25.2-27.1) [7.7-60.2]	20.7 (19.1-22.5) [6.1-47.7]
46 - 65	Male	Care home resident	72.5 (64.7-79.9) [21.4-167.1]	69 (64.6-73.7) [20.4-159]	52.7 (48.9-56.2) [15.6-121.5]	33 (30.6-35.5) [9.7-76.1]	26.1 (24-29.4) [7.7-60.3]
46 - 65	Male	Healthcare worker	53.8 (49.3-59.4) [15.9-124.1]	38.1 (37.1-39.2) [11.3-87.9]	30 (28.7-31.3) [8.9-69.1]	21.3 (19.8-22.8) [6.3-49.1]	15.6 (13.4-18.9) [4.6-35.9]
46 - 65	Male	Any comorbidity	54.3 (49.7-58.4) [16-125.2]	51.7 (50.3-53.4) [15.3-119.1]	39.5 (38.1-40.8) [11.7-91]	24.7 (23.8-25.6) [7.3-57]	19.6 (17.9-21.3) [5.8-45.2]
66 and over	Female	Baseline	75.7 (67.9-83.9) [22.4-174.6]	66.5 (64.3-68.8) [19.6-153.3]	52.3 (50.8-53.6) [15.4-120.5]	35.3 (34-36.5) [10.4-81.3]	19.9 (18.6-21.2) [5.9-45.9]
66 and over	Female	Care home resident	81.6 (72.3-90.7) [24.1-188.1]	71.6 (69.2-73.8) [21.2-165.2]	56.3 (55.2-57.7) [16.6-129.8]	38 (36.7-39.5) [11.2-87.6]	21.4 (20.3-22.9) [6.3-49.4]

66 and over	Female	Healthcare worker	48.1 (38.1-63.3) [14.2-110.9]	31.4 (26.2-37.2) [9.3-72.5]	25.4 (20.9-31.2) [7.5-58.7]	19.4 (15.6-24.3) [5.7-44.8]	10.1 (8.2-13.4) [3-23.4]
66 and over	Female	Any comorbidity	71.7 (63.8-79.6) [21.2-165.3]	63 (60.8-65.3) [18.6-145.1]	49.5 (48-51.1) [14.6-114.1]	33.4 (32.2-34.4) [9.9-77]	18.8 (17.5-20.2) [5.6-43.4]
66 and over	Male	Baseline	74 (66.6-81.4) [21.8-170.5]	64.9 (62.8-66.8) [19.2-149.7]	51.1 (49.2-52.4) [15.1-117.7]	34.4 (33.1-35.9) [10.2-79.4]	19.4 (18.3-20.7) [5.7-44.8]
66 and over	Male	Care home resident	79.7 (70.6-88.4) [23.5-183.7]	70 (67.4-72.3) [20.7-161.3]	55 (53.6-56.5) [16.2-126.8]	37.1 (35.6-38.8) [11-85.5]	20.9 (19.8-22.4) [6.2-48.3]
66 and over	Male	Healthcare worker	47 (37.3-61.4) [13.9-108.3]	30.7 (25.5-36.5) [9.1-70.8]	24.9 (20.6-30.4) [7.3-57.3]	19 (15.1-23.7) [5.6-43.8]	9.9 (7.9-13.1) [2.9-22.8]
66 and over	Male	Any comorbidity	70 (62.7-77.4) [20.7-161.4]	61.5 (59.1-63.6) [18.2-141.8]	48.3 (46.6-49.7) [14.3-111.5]	32.6 (31.3-33.8) [9.6-75.2]	18.4 (17.2-19.7) [5.4-42.4]

Appendix Table 9. Days to confirmed recovery without hospital admission, after COVID-19 onset, as in Figure 7 , by month of onset, age, sex and risk group (health care workers, care home residents, people with comorbidities, and a baseline with none of these risk factors). Times shown in days, in the following form: median (95% confidence interval for median) [95% quantiles representing variability between individuals]

Times to events following COVID-19 onset (sensitivity analysis with missing outcomes excluded)

**Age**	**Sex**	**Risk group**		**February**	**March**	**April**	**May**	**June**
45 and under	Female	Neither	No comorbidities	7.6 (7.1-8.2) [0.6-50.2]	5.5 (5.3-5.8) [0.4-36.4]	3.3 (3.1-3.6) [0.3-21.6]	1.9 (1.7-2.1) [0.2-12.5]	1.5 (1.2-1.8) [0.1-9.7]
45 and under	Female	Care home resident	No comorbidities	11.3 (10.2-13) [0.9-74.8]	8.2 (7.5-9.2) [0.7-54.2]	4.9 (4.5-5.5) [0.4-32.2]	2.8 (2.4-3.4) [0.2-18.6]	2.2 (1.8-2.8) [0.2-14.5]
45 and under	Female	Healthcare worker	No comorbidities	6.6 (6-7.6) [0.5-43.7]	4.8 (4.4-5.3) [0.4-31.6]	2.9 (2.6-3.2) [0.2-18.8]	1.6 (1.4-1.9) [0.1-10.9]	1.3 (1-1.6) [0.1-8.5]
45 and under	Female	Neither	Comorbidity	6.9 (6.3-7.5) [0.6-45.4]	5 (4.6-5.3) [0.4-32.9]	3 (2.8-3.2) [0.2-19.6]	1.7 (1.5-1.9) [0.1-11.3]	1.3 (1.1-1.7) [0.1-8.8]
45 and under	Male	Neither	No comorbidities	7.6 (7-8.1) [0.6-49.9]	5.5 (5.2-5.8) [0.4-36.1]	3.3 (3.1-3.5) [0.3-21.5]	1.9 (1.7-2.1) [0.2-12.4]	1.5 (1.2-1.8) [0.1-9.7]
45 and under	Male	Care home resident	No comorbidities	11.3 (10.1-13) [0.9-74.3]	8.2 (7.3-9.2) [0.7-53.8]	4.9 (4.5-5.5) [0.4-32]	2.8 (2.4-3.4) [0.2-18.5]	2.2 (1.8-2.7) [0.2-14.4]
45 and under	Male	Healthcare worker	No comorbidities	6.6 (5.9-7.4) [0.5-43.4]	4.8 (4.4-5.4) [0.4-31.4]	2.8 (2.6-3.2) [0.2-18.7]	1.6 (1.4-1.9) [0.1-10.8]	1.3 (1-1.6) [0.1-8.4]
45 and under	Male	Neither	Comorbidity	6.8 (6.3-7.4) [0.6-45.1]	5 (4.7-5.3) [0.4-32.7]	2.9 (2.8-3.2) [0.2-19.4]	1.7 (1.5-1.9) [0.1-11.2]	1.3 (1.1-1.7) [0.1-8.7]

46 - 65	Female	Neither	No comorbidities	8.6 (8-9.3) [0.7-56.5]	6.2 (5.9-6.5) [0.5-41]	3.7 (3.5-4) [0.3-24.4]	2.1 (1.9-2.4) [0.2-14.1]	1.7 (1.3-2.1) [0.1-11]
46 - 65	Female	Care home resident	No comorbidities	12.8 (11.4-14.6) [1-84.2]	9.2 (8.6-10.4) [0.8-61]	5.5 (5.2-6.2) [0.4-36.3]	3.2 (2.7-3.7) [0.3-21]	2.5 (2-3.1) [0.2-16.3]
46 - 65	Female	Healthcare worker	No comorbidities	7.5 (6.7-8.5) [0.6-49.2]	5.4 (4.9-5.9) [0.4-35.6]	3.2 (2.9-3.5) [0.3-21.2]	1.9 (1.6-2.1) [0.2-12.3]	1.4 (1.1-1.8) [0.1-9.5]
46 - 65	Female	Neither	Comorbidity	7.7 (7.1-8.5) [0.6-51.1]	5.6 (5.3-5.9) [0.5-37]	3.3 (3.1-3.6) [0.3-22]	1.9 (1.7-2.2) [0.2-12.7]	1.5 (1.2-1.9) [0.1-9.9]
46 - 65	Male	Neither	No comorbidities	8.5 (8-9.2) [0.7-56.2]	6.2 (6-6.5) [0.5-40.7]	3.7 (3.5-3.9) [0.3-24.2]	2.1 (1.9-2.4) [0.2-14]	1.7 (1.3-2.1) [0.1-10.9]
46 - 65	Male	Care home resident	No comorbidities	12.7 (11.5-14.7) [1-83.7]	9.2 (8.5-10.5) [0.7-60.7]	5.5 (5.1-6.2) [0.4-36.1]	3.2 (2.7-3.7) [0.3-20.9]	2.5 (2-3) [0.2-16.2]
46 - 65	Male	Healthcare worker	No comorbidities	7.4 (6.7-8.4) [0.6-48.9]	5.4 (4.9-5.9) [0.4-35.4]	3.2 (2.9-3.5) [0.3-21.1]	1.8 (1.5-2.1) [0.2-12.2]	1.4 (1.1-1.8) [0.1-9.5]
46 - 65	Male	Neither	Comorbidity	7.7 (7.2-8.3) [0.6-50.8]	5.6 (5.3-5.8) [0.5-36.8]	3.3 (3.2-3.6) [0.3-21.9]	1.9 (1.7-2.2) [0.2-12.7]	1.5 (1.2-1.9) [0.1-9.8]
66 and over	Female	Neither	No comorbidities	7 (6.6-7.6) [0.6-46.3]	5.1 (4.9-5.4) [0.4-33.5]	3 (2.9-3.3) [0.2-20]	1.7 (1.6-1.9) [0.1-11.5]	1.4 (1.1-1.6) [0.1-9]
66 and over	Female	Care home resident	No comorbidities	10.5 (9.3-12) [0.8-68.9]	7.6 (7-8.4) [0.6-49.9]	4.5 (4.2-5) [0.4-29.7]	2.6 (2.2-3) [0.2-17.2]	2 (1.6-2.5) [0.2-13.4]
66 and over	Female	Healthcare worker	No comorbidities	6.1 (5.5-6.9) [0.5-40.3]	4.4 (4-4.9) [0.4-29.2]	2.6 (2.4-2.9) [0.2-17.4]	1.5 (1.3-1.7) [0.1-10]	1.2 (0.9-1.4) [0.1-7.8]
66 and over	Female	Neither	Comorbidity	6.3 (6-6.8) [0.5-41.8]	4.6 (4.4-4.8) [0.4-30.3]	2.7 (2.6-2.9) [0.2-18]	1.6 (1.4-1.8) [0.1-10.4]	1.2 (1-1.5) [0.1-8.1]
66 and over	Male	Neither	No comorbidities	7 (6.5-7.5) [0.6-46]	5.1 (4.8-5.3) [0.4-33.3]	3 (2.9-3.3) [0.2-19.8]	1.7 (1.5-1.9) [0.1-11.5]	1.4 (1.1-1.6) [0.1-8.9]
66 and over	Male	Care home resident	No comorbidities	10.4 (9.3-12) [0.8-68.5]	7.5 (6.9-8.5) [0.6-49.7]	4.5 (4.1-5) [0.4-29.5]	2.6 (2.2-3) [0.2-17.1]	2 (1.6-2.5) [0.2-13.3]
66 and over	Male	Healthcare worker	No comorbidities	6.1 (5.5-6.9) [0.5-40]	4.4 (4-4.9) [0.4-29]	2.6 (2.4-2.9) [0.2-17.3]	1.5 (1.3-1.7) [0.1-10]	1.2 (0.9-1.5) [0.1-7.8]
66 and over	Male	Neither	Comorbidity	6.3 (6-6.7) [0.5-41.6]	4.6 (4.4-4.7) [0.4-30.1]	2.7 (2.6-2.9) [0.2-17.9]	1.6 (1.4-1.8) [0.1-10.4]	1.2 (1-1.5) [0.1-8.1]

Appendix Table 10. Days to hospital admission after COVID-19 onset, by month of onset, age, sex and risk group (health care workers, care home residents, people with comorbidities, and a baseline with none of these risk factors). Times shown in days, in the following form: median (95% confidence interval for median) [95% quantiles representing variability between individuals]. Sensitivity analysis with missing outcomes excluded.

Age	Sex	Risk group		February	March	April	May	June
45 and under	Female	Neither	No comorbidities	21 (12.6-33) [1.5-106.3]	9.4 (7.2-13.8) [0.7-47.7]	8.8 (6.8-12.9) [0.6-44.5]	9.1 (6.2-13.5) [0.6-45.8]	9.1 (6.2-13.5) [0.6-45.8]
45 and under	Female	Care home resident	No comorbidities	30.2 (17.4-48) [2.1-153]	13.6 (10-18.9) [1-68.7]	12.7 (9.4-17.8) [0.9-64.1]	13 (8.9-20.1) [0.9-65.9]	13 (8.9-20.1) [0.9-65.9]
45 and under	Female	Healthcare worker	No comorbidities	21 (12.6-33) [1.5-106.3]	9.4 (7.2-13.8) [0.7-47.7]	8.8 (6.8-12.9) [0.6-44.5]	9.1 (6.2-13.5) [0.6-45.8]	9.1 (6.2-13.5) [0.6-45.8]
45 and under	Female	Neither	Comorbidity	20.2 (11.7-31.5) [1.4-102.1]	9.1 (6.7-13.3) [0.6-45.8]	8.5 (6.2-12) [0.6-42.8]	8.7 (5.9-12.2) [0.6-44]	8.7 (5.9-12.2) [0.6-44]
45 and under	Male	Neither	No comorbidities	19.1 (11.5-29.2) [1.4-96.6]	8.6 (6.6-11.8) [0.6-43.4]	8 (6.1-11.1) [0.6-40.5]	8.2 (5.8-11.3) [0.6-41.6]	8.2 (5.8-11.3) [0.6-41.6]
45 and under	Male	Care home resident	No comorbidities	27.5 (16-42.2) [1.9-139]	12.3 (9.4-16.8) [0.9-62.4]	11.5 (9-16) [0.8-58.2]	11.8 (8.4-17) [0.8-59.9]	11.8 (8.4-17) [0.8-59.9]
45 and under	Male	Healthcare worker	No comorbidities	19.1 (11.5-29.2) [1.4-96.6]	8.6 (6.6-11.8) [0.6-43.4]	8 (6.1-11.1) [0.6-40.5]	8.2 (5.8-11.3) [0.6-41.6]	8.2 (5.8-11.3) [0.6-41.6]
45 and under	Male	Neither	Comorbidity	18.3 (10.7-28.9) [1.3-92.8]	8.2 (6-11.5) [0.6-41.6]	7.7 (5.6-10.6) [0.5-38.9]	7.9 (5.5-10.6) [0.6-40]	7.9 (5.5-10.6) [0.6-40]
46 - 65	Female	Neither	No comorbidities	21 (12.6-33) [1.5-106.3]	9.4 (7.2-13.8) [0.7-47.7]	8.8 (6.8-12.9) [0.6-44.5]	9.1 (6.2-13.5) [0.6-45.8]	9.1 (6.2-13.5) [0.6-45.8]
46 - 65	Female	Care home resident	No comorbidities	30.2 (17.4-48) [2.1-153]	13.6 (10-18.9) [1-68.7]	12.7 (9.4-17.8) [0.9-64.1]	13 (8.9-20.1) [0.9-65.9]	13 (8.9-20.1) [0.9-65.9]
46 - 65	Female	Healthcare worker	No comorbidities	21 (12.6-33) [1.5-106.3]	9.4 (7.2-13.8) [0.7-47.7]	8.8 (6.8-12.9) [0.6-44.5]	9.1 (6.2-13.5) [0.6-45.8]	9.1 (6.2-13.5) [0.6-45.8]
46 - 65	Female	Neither	Comorbidity	20.2 (11.7-31.5) [1.4-102.1]	9.1 (6.7-13.3) [0.6-45.8]	8.5 (6.2-12) [0.6-42.8]	8.7 (5.9-12.2) [0.6-44]	8.7 (5.9-12.2) [0.6-44]
46 - 65	Male	Neither	No comorbidities	19.1 (11.5-29.2) [1.4-96.6]	8.6 (6.6-11.8) [0.6-43.4]	8 (6.1-11.1) [0.6-40.5]	8.2 (5.8-11.3) [0.6-41.6]	8.2 (5.8-11.3) [0.6-41.6]
46 - 65	Male	Care home resident	No comorbidities	27.5 (16-42.2) [1.9-139]	12.3 (9.4-16.8) [0.9-62.4]	11.5 (9-16) [0.8-58.2]	11.8 (8.4-17) [0.8-59.9]	11.8 (8.4-17) [0.8-59.9]
46 - 65	Male	Healthcare worker	No comorbidities	19.1 (11.5-29.2) [1.4-96.6]	8.6 (6.6-11.8) [0.6-43.4]	8 (6.1-11.1) [0.6-40.5]	8.2 (5.8-11.3) [0.6-41.6]	8.2 (5.8-11.3) [0.6-41.6]
46 - 65	Male	Neither	Comorbidity	18.3 (10.7-28.9) [1.3-92.8]	8.2 (6-11.5) [0.6-41.6]	7.7 (5.6-10.6) [0.5-38.9]	7.9 (5.5-10.6) [0.6-40]	7.9 (5.5-10.6) [0.6-40]
66 and over	Female	Neither	No comorbidities	23.8 (15.2-33.2) [1.7-120.4]	10.7 (9.4-12.1) [0.8-54.1]	10 (8.7-11.6) [0.7-50.5]	10.3 (7.8-13.1) [0.7-51.9]	10.3 (7.8-13.1) [0.7-51.9]

66 and over	Female	Care home resident	No comorbidities	34.3 (22-47.1) [2.4-173.3]	15.4 (13.8-17.5) [1.1-77.8]	14.4 (13.1-16) [1-72.6]	14.8 (11.8-17.5) [1-74.7]	14.8 (11.8-17.5) [1-74.7]
66 and over	Female	Healthcare worker	No comorbidities	23.8 (15.2-33.2) [1.7-120.4]	10.7 (9.4-12.1) [0.8-54.1]	10 (8.7-11.6) [0.7-50.5]	10.3 (7.8-13.1) [0.7-51.9]	10.3 (7.8-13.1) [0.7-51.9]
66 and over	Female	Neither	Comorbidity	22.9 (14.3-30.8) [1.6-115.7]	10.3 (9.1-11.5) [0.7-51.9]	9.6 (8.4-11.1) [0.7-48.5]	9.9 (7.7-12.2) [0.7-49.8]	9.9 (7.7-12.2) [0.7-49.8]
66 and over	Male	Neither	No comorbidities	21.6 (14.5-29.6) [1.5-109.5]	9.7 (8.4-11.1) [0.7-49.1]	9.1 (7.8-10.4) [0.6-45.9]	9.3 (7.3-11.8) [0.7-47.2]	9.3 (7.3-11.8) [0.7-47.2]
66 and over	Male	Care home resident	No comorbidities	31.1 (20.7-42.6) [2.2-157.5]	14 (12-16) [1-70.7]	13 (11.4-14.8) [0.9-66]	13.4 (11-16.1) [0.9-67.9]	13.4 (11-16.1) [0.9-67.9]
66 and over	Male	Healthcare worker	No comorbidities	21.6 (14.5-29.6) [1.5-109.5]	9.7 (8.4-11.1) [0.7-49.1]	9.1 (7.8-10.4) [0.6-45.9]	9.3 (7.3-11.8) [0.7-47.2]	9.3 (7.3-11.8) [0.7-47.2]
66 and over	Male	Neither	Comorbidity	20.8 (13.6-28.6) [1.5-105.1]	9.3 (8.4-10.3) [0.7-47.2]	8.7 (7.6-9.7) [0.6-44]	9 (7.1-11) [0.6-45.3]	9 (7.1-11) [0.6-45.3]

Appendix Table 11. Days to death before hospital admission, after COVID-19 onset, by month of onset, age, sex and risk group (health care workers, care home residents, people with comorbidities, and a baseline with none of these risk factors). Times shown in days, in the following form: median (95% confidence interval for median) [95% quantiles representing variability between individuals].Sensitivity analysis with missing outcomes excluded.

Age	Sex	Risk group		February	March	April	May	June
45 and under	Female	Neither	No comorbidities	47 (41.7-53.8) [10.1-131.4]	53.3 (51.9-54.7) [16.3-125.4]	37 (35.6-38.1) [9.6-94.9]	22.8 (21.7-23.5) [7.4-51.8]	17.3 (16.5-18.3) [7.7-32.9]
45 and under	Female	Care home resident	No comorbidities	43.1 (35-51.8) [9.3-120.4]	48.9 (41.3-57.7) [14.9-115]	33.9 (28.3-39.6) [8.8-87]	20.9 (17.5-24.3) [6.8-47.4]	15.8 (13.3-18.8) [7-30.1]
45 and under	Female	Healthcare worker	No comorbidities	45.3 (39-52.2) [9.7-126.7]	39.3 (38.1-40.4) [12-92.5]	28.7 (27.3-30.2) [7.4-73.6]	20.8 (19-22.3) [6.8-47.2]	16.2 (14.3-19.1) [7.2-30.8]
45 and under	Female	Neither	Comorbidity	45.5 (40.2-51.6) [9.8-127.4]	51.7 (50.1-53.3) [15.8-121.6]	35.9 (34.3-37.2) [9.3-92]	22.1 (20.9-22.8) [7.2-50.2]	16.8 (15.9-17.8) [7.4-31.9]
45 and under	Male	Neither	No comorbidities	44.7 (39.5-51.2) [9.6-125.1]	50.8 (49.4-52.1) [15.5-119.4]	35.2 (33.9-36.4) [9.1-90.3]	21.7 (20.6-22.4) [7-49.3]	16.5 (15.7-17.4) [7.3-31.3]
45 and under	Male	Care home resident	No comorbidities	41 (33.3-50) [8.8-114.7]	46.5 (39.1-54.9) [14.2-109.5]	32.3 (26.8-37.7) [8.3-82.8]	19.9 (16.5-23.1) [6.5-45.2]	15.1 (12.5-17.9) [6.7-28.7]
45 and under	Male	Healthcare worker	No comorbidities	43.1 (37.3-49.9) [9.3-120.6]	37.5 (36.2-38.6) [11.4-88.1]	27.3 (25.9-28.9) [7.1-70.1]	19.8 (18.2-21.1) [6.4-45]	15.4 (13.5-18.2) [6.8-29.3]
45 and under	Male	Neither	Comorbidity	43.4 (38.3-49.2) [9.3-121.3]	49.2 (47.7-50.7) [15-115.8]	34.2 (32.7-35.3) [8.8-87.6]	21 (19.9-21.8) [6.8-47.8]	16 (15.1-16.9) [7.1-30.3]
46 - 65	Female	Neither	No comorbidities	54.5 (48.9-58.5) [11.8-151.5]	53.6 (52.3-55) [18-119.7]	39.3 (38.1-40.6) [11.6-94.2]	23.1 (21.9-24.2) [7.5-52.6]	17.4 (16.4-18.5) [8.1-32.1]
46 - 65	Female	Care home resident	No comorbidities	67.8 (58.7-74.9) [14.7-188.5]	66.7 (61.9-70.6) [22.4-148.9]	48.8 (45.4-52.1) [14.4-117.1]	28.8 (26.5-30.5) [9.4-65.4]	21.7 (19.7-23.5) [10-40]
46 - 65	Female	Healthcare worker	No comorbidities	50.9 (45-55.1) [11.1-141.5]	38.3 (37.5-39.5) [12.9-85.5]	29.5 (28.4-30.8) [8.7-70.7]	20.4 (18.7-22) [6.7-46.5]	15.8 (14-18.7) [7.3-29.2]
46 - 65	Female	Neither	Comorbidity	52.8 (47.5-56.9) [11.5-146.9]	52 (50.4-53.7) [17.5-116]	38.1 (36.9-39.8) [11.2-91.3]	22.4 (21-23.5) [7.3-51]	16.9 (15.8-18.1) [7.8-31.2]
46 - 65	Male	Neither	No comorbidities	51.9 (46.3-55.9) [11.3-144.3]	51 (49.7-52.4) [17.2-113.9]	37.4 (36.4-38.7) [11-89.7]	22 (20.9-23.1) [7.2-50.1]	16.6 (15.7-17.5) [7.7-30.6]
46 - 65	Male	Care home resident	No comorbidities	64.5 (56-70.9) [14-179.4]	63.5 (59.3-67.1) [21.4-141.7]	46.5 (43-49.4) [13.7-111.5]	27.4 (25.1-29) [8.9-62.3]	20.6 (18.9-22.2) [9.6-38.1]
46 - 65	Male	Healthcare worker	No comorbidities	48.5 (42.9-52.6) [10.5-134.7]	36.5 (35.5-37.7) [12.3-81.4]	28.1 (27-29.3) [8.3-67.4]	19.5 (17.8-20.9) [6.3-44.2]	15 (13.5-17.8) [7-27.8]
46 - 65	Male	Neither	Comorbidity	50.3 (44.9-53.9) [10.9-139.9]	49.5 (48-51) [16.7-110.5]	36.3 (35.1-37.8) [10.7-86.9]	21.4 (20.1-22.5) [7-48.5]	16.1 (15.1-17.1) [7.5-29.7]
66 and over	Female	Neither	No comorbidities	73 (64.7-79.2) [27-154.5]	60.4 (58.5-62.3) [21.4-131.1]	47.2 (45.7-48.6) [19.3-94.2]	28.9 (27.3-30.1) [7.9-72]	15.8 (14.8-16.8) [5.8-33.5]

66 and over	Female	Care home resident	No comorbidities	81.1 (71.5-89.1) [30-171.6]	67.1 (65.1-69.2) [23.8-145.6]	52.4 (51.4-53.7) [21.4-104.6]	32.1 (30.7-33.1) [8.8-79.9]	17.5 (16.4-18.7) [6.5-37.2]
66 and over	Female	Healthcare worker	No comorbidities	49.9 (39.2-67.7) [18.5-105.6]	31.6 (26.9-39.1) [11.2-68.5]	26 (21.9-32.4) [10.6-51.8]	18.7 (15.4-23) [5.1-46.5]	10.5 (8.4-13.9) [3.9-22.3]
66 and over	Female	Neither	Comorbidity	70.8 (63.1-77) [26.2-149.8]	58.6 (56.4-60.7) [20.8-127.1]	45.8 (44.3-46.9) [18.7-91.3]	28 (26.6-29.2) [7.7-69.8]	15.3 (14.4-16.2) [5.6-32.5]
66 and over	Male	Neither	No comorbidities	69.5 (61.2-75.4) [25.7-147.1]	57.5 (55.4-59.6) [20.4-124.8]	44.9 (43.5-46.4) [18.4-89.7]	27.5 (26.1-28.7) [7.5-68.5]	15 (14.2-15.9) [5.5-31.9]
66 and over	Male	Care home resident	No comorbidities	77.2 (67.8-84.9) [28.6-163.4]	63.9 (62-66.4) [22.7-138.6]	49.9 (48.7-51.1) [20.4-99.6]	30.6 (29.2-31.7) [8.4-76.1]	16.7 (15.8-17.7) [6.1-35.5]
66 and over	Male	Healthcare worker	No comorbidities	47.5 (37.3-64.9) [17.6-100.6]	30.1 (25.5-37.1) [10.7-65.3]	24.7 (20.9-30.7) [10.1-49.3]	17.8 (14.7-22) [4.9-44.3]	10 (8-13.3) [3.7-21.2]
66 and over	Male	Neither	Comorbidity	67.4 (59.8-73.5) [24.9-142.6]	55.8 (53.7-57.8) [19.8-121]	43.6 (42.2-44.6) [17.8-87]	26.7 (25.2-27.9) [7.3-66.4]	14.6 (13.7-15.3) [5.4-31]

Appendix Table 12. Days to confirmed recovery without hospital admission, after COVID-19 onset, by month of onset, age, sex and risk group (health care workers, care home residents, people with comorbidities, and a baseline with none of these risk factors). Times shown in days, in the following form: median (95% confidence interval for median) [95% quantiles representing variability between individuals]. Sensitivity analysis with missing outcomes excluded.

Details of selected regression models for predictors of outcomes after onset

The multi-state mixture model is composed of two parts: one part determining the event that happens next, and another part determining the time to that event for each potential event. In the first part, the odds of admission and death (without admission) are defined as different log-linear functions of covariates, where the odds of admission or death is the probability of admission or death respectively divided by the probability of recovery. In the second part, the time to each event was assumed to be distributed as a generalized gamma, a flexible three-parameter distribution that is defined in terms of covariates through an accelerated failure time model (Jackson 2018). In both parts, a “best-fitting” dependence on covariates was determined from a range of choices by minimising Akaike’s information criterion. This range included models containing interactions of all other covariates with age and month of onset. The goodness-of-fit of the selected models was verified against the observed data by comparing against nonparametric estimates (Appendix Figures 8-10).

The tables below have one row showing the log odds ratio (and standard error) relating to each main effect or interaction term in the selected regression model, thus showing which terms define the model. These are presented here for technical completeness and to permit comparison of the models with and without censored data. (Note that a clearer presentation of the results of the regression model is given by comparing the absolute probabilities of outcomes between different groups, as in e.g. Appendix Tables 3,4).

The log odds ratios are interpreted as follows. A "baseline" group is defined as age 45, female, onset in March, no comorbidities, not a care home resident or healthcare worker. The log odds ratio comparing a group of interest with the baseline group can be computed by summing each term relating to the group, e.g. the log odds ratio for a healthcare worker aged 66+, relative to the baseline group, should be computed by adding the "Healthcare worker" main effect to the "Healthcare worker, age 66+" interaction term.

	**Excluding censoring**	**Including censoring**
Onset in February	0.57 (0.16)	0.52 (0.15)
Onset in April	-0.44 (0.08)	-0.43 (0.08)
Onset in May	-0.92 (0.13)	-0.93 (0.13)
Onset in June	-1.23 (0.22)	-1.49 (0.21)
Age 46-65	0.62 (0.07)	0.62 (0.07)
Age 66+	1.95 (0.09)	1.77 (0.08)
Male	0.52 (0.07)	0.43 (0.07)
Care home resident	-1.59 (0.42)	-1.71 (0.41)
Healthcare worker	-1.53 (0.09)	-1.45 (0.09)
Any comorbidity	0.55 (0.05)	0.58 (0.05)
Onset in February, age 46-65	-0.05 (0.18)	-0.03 (0.17)
Onset in April, age 46-65	-0.46 (0.1)	-0.48 (0.1)
Onset in May, age 46-65	-0.94 (0.16)	-0.91 (0.16)

Onset in June, age 46-65	-0.85 (0.31)	-0.75 (0.29)
Onset in February, age 66+	-0.09 (0.23)	-0.03 (0.22)
Onset in April, age 66+	-1.07 (0.12)	-1.07 (0.11)
Onset in May, age 66+	-1.03 (0.17)	-0.87 (0.17)
Onset in June, age 66+	-1.01 (0.28)	-0.73 (0.27)
Age 46-65, male	0.47 (0.08)	0.53 (0.08)
Male, age 66+	0.19 (0.08)	0.28 (0.08)
Age 46-65, care home resident	-0.38 (0.45)	-0.35 (0.44)
Age 66+, care home resident	-1.38 (0.42)	-1.18 (0.41)
Age 46-65, healthcare worker	-0.36 (0.11)	-0.37 (0.11)
Age 66+, healthcare worker	-0.25 (0.35)	-0.15 (0.35)
Onset in February, male	-0.21 (0.14)	-0.16 (0.14)
Onset in April, male	0.03 (0.07)	0.02 (0.07)
Onset in May, male	-0.19 (0.13)	-0.18 (0.13)
Onset in June, male	-0.65 (0.23)	-0.61 (0.22)
Onset in February, care home resident	0.47 (0.42)	0.5 (0.41)
Onset in April, care home resident	0.82 (0.12)	0.81 (0.11)
Onset in May, care home resident	0.61 (0.21)	0.52 (0.21)
Onset in June, care home resident	1.59 (0.43)	1.57 (0.42)
Onset in February, any comorbidity	-0.14 (0.17)	-0.05 (0.16)
Onset in April, any comorbidity	-0.08 (0.08)	-0.05 (0.08)
Onset in May, any comorbidity	-0.43 (0.16)	-0.43 (0.16)
Onset in June, any comorbidity	1.08 (1.28)	1.26 (1.28)

Appendix Table 13. Log odds ratios from model for predictors of hospital admission. Odds defined as probability of admission divided by probability of recovery without admission.

	**Excluding censoring**	**Including censoring**
Onset in February	-0.9 (0.3)	-0.8 (0.29)
Onset in April	-0.88 (0.12)	-0.85 (0.12)
Onset in May or June	-2.53 (0.29)	-2.41 (0.29)
Age 66+	5.34 (0.3)	5.19 (0.3)
Male	0.33 (0.07)	0.33 (0.07)
Care home resident	3.01 (0.38)	2.9 (0.38)
Any comorbidity	1.36 (0.37)	1.44 (0.36)
Age 66+, care home resident	-3.06 (0.39)	-2.88 (0.38)

Age 66+, any comorbidity	-1.06 (0.37)	-1.11 (0.37)
Onset in Februrary, care home resident	0.37 (0.6)	0.4 (0.59)
Onset in April, care home resident	0.26 (0.15)	0.25 (0.14)
Onset in May or June, care home resident	0.7 (0.33)	0.62 (0.33)

Appendix Table 14. Log odds ratios from model for predictors of death before hospital admission. Odds defined as probability of death before admission divided by probability of recovery without admission. (Note: as there were no deaths from onsets in June, the odds rat io for death following onset in June was constrained to be equal to that for May. Due to the small numbers of deaths in the two age groups below 65, these groups were merged. No deaths before admission were observed among healthcare workers, so this predictor was excluded.)

	**Excluding censoring**	**Including censoring**
Age 46-65	-0.12 (0.03)	-0.12 (0.03)
Age 66+	0.08 (0.03)	0.07 (0.04)
Male	0.01 (0.02)	0 (0.02)
Onset in February	-0.32 (0.03)	-0.32 (0.03)
Onset in April	0.52 (0.03)	0.53 (0.03)
Onset in May	1.07 (0.06)	1.08 (0.06)
Onset in June	1.32 (0.11)	1.25 (0.11)
Healthcare worker	0.14 (0.05)	0.15 (0.05)
Care home resident	-0.4 (0.05)	-0.38 (0.05)
Any comorbidity	0.1 (0.02)	0.1 (0.02)

Appendix Table 15. Predictors in selected model for time from COVID-19 onset to hospital admission. Each row represents a covariate effect (main effect or interaction term), defined as the ratio of the expected time to admission with, versus without, the indicated covariate.

	**Excluding censoring**	**Including censoring**
Age 66+	-0.12 (0.18)	-0.15 (0.17)
Male	0.1 (0.06)	0.09 (0.06)
Onset in February	-0.8 (0.22)	-0.79 (0.22)
Onset in April	0.07 (0.06)	0.07 (0.06)
Onset in May or June	0.04 (0.12)	0.04 (0.12)
Care home resident	-0.36 (0.06)	-0.36 (0.06)
Any comorbidity	0.04 (0.06)	0.04 (0.06)

Appendix Table 16. Predictors in selected model for time from COVID-19 onset to death without hospital admission. Each row represents a covariate effect (main effect or interaction term), defined as the ratio of the expected time to death with, versus without, the indicated covariate.

	**Excluding censoring**	**Including censoring**
Any comorbidity	-0.03 (0.01)	-0.05 (0.01)
Male	-0.05 (0.01)	-0.02 (0.01)
Onset in February	0.31 (0.12)	-0.06 (0.04)
Onset in April	-0.14 (0.06)	-0.3 (0.02)
Onset in May	-0.95 (0.07)	-0.69 (0.02)
Onset in June	-1.78 (0.12)	-0.87 (0.03)
Healthcare worker	-0.3 (0.02)	-0.32 (0.02)
Care home resident	-0.09 (0.09)	0.02 (0.07)
Age 46-65	-0.14 (0.04)	-0.02 (0.01)
Age 66+	-0.11 (0.06)	0.15 (0.02)
Onset in February (age 46-65)	0.28 (0.15)	0.11 (0.06)
Onset in April (age 46-65)	0.03 (0.08)	0.03 (0.02)
Onset in May (age 46-65)	0.16 (0.09)	-0.05 (0.03)
Onset in June (age 46-65)	0.04 (0.19)	-0.1 (0.05)
Onset in February (age 66+)	-0.2 (0.2)	0.19 (0.07)
Onset in April (age 66+)	-0.37 (0.08)	0.06 (0.02)
Onset in May (age 66+)	0.59 (0.1)	0.05 (0.03)
Onset in June (age 66+)	0.38 (0.15)	-0.34 (0.05)
Healthcare worker, age 46-65	-0.03 (0.02)	-0.04 (0.02)
Healthcare worker, age 66+	-0.34 (0.1)	-0.43 (0.11)
Care home resident, age 46-65	0.31 (0.1)	0.22 (0.08)
Care home resident, age 66+	0.19 (0.09)	0.06 (0.07)
Onset in February (healthcare worker)	0.27 (0.07)	0.3 (0.06)
Onset in April (healthcare worker)	0.05 (0.03)	0.03 (0.03)
Onset in May (healthcare worker)	0.21 (0.04)	0.15 (0.04)
Onset in June (healthcare worker)	0.24 (0.07)	0.07 (0.1)

Appendix Table 17. Predictors in selected model for time from COVID-19 onset to confirmed recovery (second of two negative tests). Each row represents a covariate effect (main effect or interaction term), defined as the ratio of the expected time to confirmed recovery with, versus without, the indicated covariate.

Goodness of fit of selected parametric models

The overall fit of the parametric assumptions of the multi-state model is checked by comparing predictions of the cumulative incidence probability of events from the parametric model with estimates from the nonparametric Aalen-Johansen method. These parametric assumptions include the form of the distribution for the time to the next event after onset, the selection of covariates that affect the parameters of this distribution, and the selection of covariates that affect the probability governing which of the next event happens. These checks are illustrated in Appendix Figures 8-10. The plots also compare the AIC-selected models fitted to the data (a) with the missing outcomes ignored, and (b) with the missing outcomes considered as censoring.

The parametric estimates only deviate from the nonparametric estimates in cases where the amount of data informing the nonparametric estimate is small, thus the nonparametric estimate is unreliable. This includes the probability of admission versus recovery beyond 20 days after onset for males aged 45 and below with onset in February (bottom row of Appendix Figure 8), where there were 46 admissions for people in this group, and only three of these beyond 20 days after onset. In Appendix Figure 9, while the fit appears to be worst for healthcare workers aged over 65 with onset in March, and care home residents under 65 with onset in June, these categories included only 33 and 3 observed cases respectively.

In Appendix Figure 10, note that the nonparametric estimates could not be calculated for certain combinations of predictors where there were no or very few observations (e.g. onset in June for people with comorbidities). In these cases, the parametric models allow prediction through the assumption of additivity of terms of the regression model.

Appendix Figure 11 shows the fit of modelled densities to data. This shows parametrically modelled densities for a “baseline” category defined by female, no comorbidities, not a care home resident or healthcare worker, and compares three age groups, for the three different competing events following onset. The model accounting for censoring is compared with the model that neglects the censored data. For the times from onset to hospital admission and death, these two models agree, since if a patient had not died or been admitted to hospital by the end of their follow-up period, they are judged likely to recover. Hence there is negligible censoring of admission or death events, and some censoring of recovery events. Therefore for the event of recovery, the model that accounts for censoring predicts slightly longer times to events, and we would judge that the model more accurately reflects the true distribution for this event.

Supplementary analysis with a finer age categorisation

An additional model was developed to investigate the relation of age and care home residency to mortality before hospital admission in more detail. For this model, a finer age grouping was defined by splitting the single over-65s group into three categories: ages 65-74, ages 75-84 and ages 85 and over. A logistic regression model was fitted to the uncensored data. Selected covariates included age group, month of admission, gender, care home residency, comorbidities, and an interaction of gender and care home residency with age group and month of admission. Estimated probabilities of death before admission, by age group, gender and care home residency, for a baseline group defined by people

without comorbidities and onset in March, are presented in Appendix Table 18. Within each age and gender group, people in care homes had a substantially higher risk of death before admission.

Non care-home residents		
	Men	Women
Age 0-45	0 (0, 0.01)	0 (0, 0.01)
Age 46-65	0 (0, 0)	0 (0, 0.01)
Age 66-75	0.03 (0.02, 0.04)	0.02 (0.01, 0.03)
Age 76-85	0.06 (0.05, 0.08)	0.06 (0.04, 0.07)
Age 86+	0.15 (0.12, 0.18)	0.12 (0.1, 0.15)
Care-home residents		
Age 0-45	0.03 (0, 0.23)	0.07 (0.01, 0.43)
Age 46-65	0.04 (0.02, 0.09)	0.09 (0.04, 0.19)
Age 66-75	0.22 (0.15, 0.3)	0.17 (0.11, 0.25)
Age 76-85	0.26 (0.21, 0.32)	0.24 (0.19, 0.29)
Age 86+	0.36 (0.3, 0.42)	0.31 (0.27, 0.36)

Appendix Table 18. Estimated probabilities of death before hospital admission by age group (using a finer classification) and care home residency, for people without comorbidities and onset in March 2020.

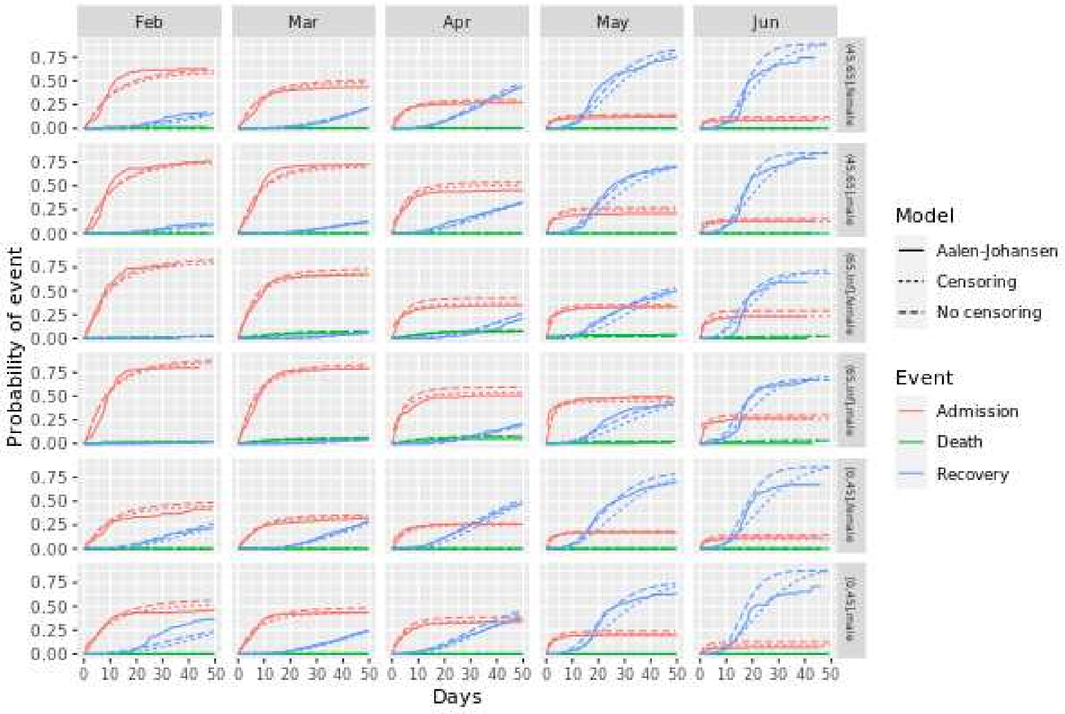

Appendix Figure 8. Predicted cumulative incidence of next events following COVID-19 onset, for the selected parametric models with and without censoring, compared to Aalen-Johansen nonparametric estimates to check goodness of fit. By month of onset, age and gender.

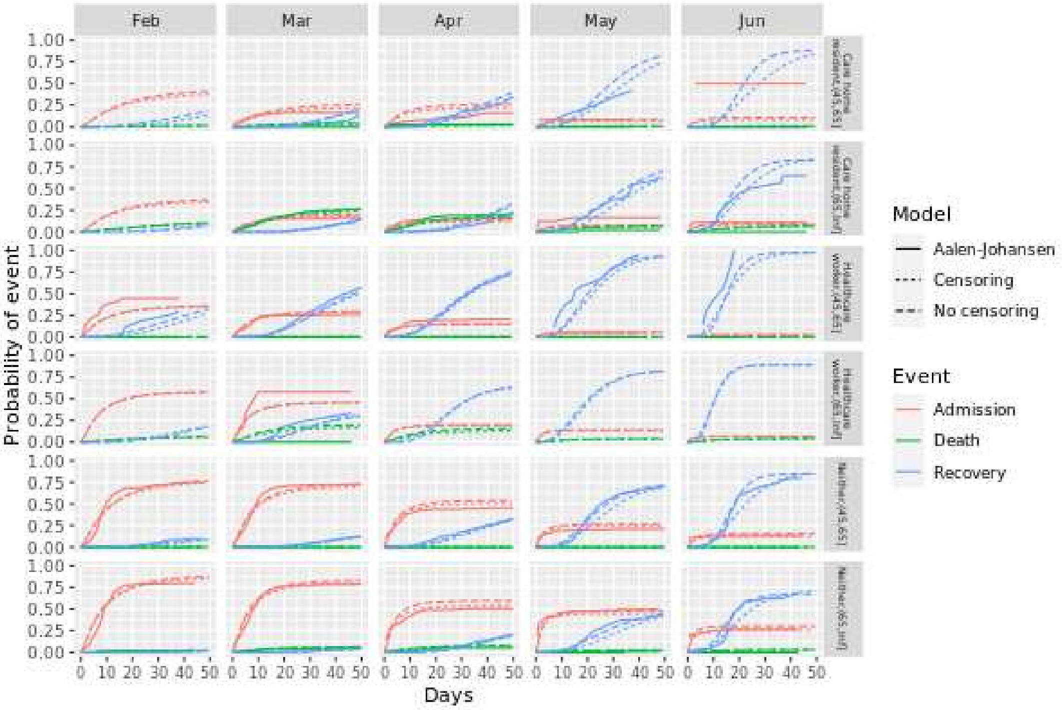

Appendix Figure 9. Predicted cumulative incidence of next events following COVID-19 onset, for the selected parametric models with and without censoring, compared to Aalen-Johansen nonparametric estimates to check goodness of fit. By month of onset and age group (for men, excluding under-45s and people with comorbidities), comparing care home residents and healthcare workers with people who were neither.

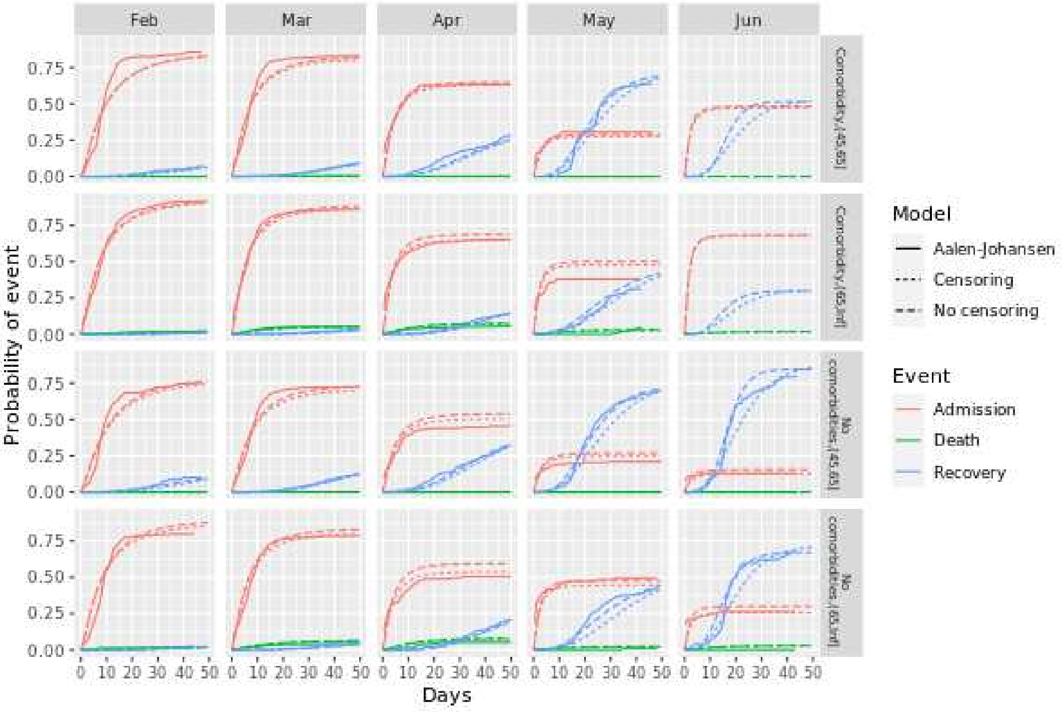

Appendix Figure 10. Predicted cumulative incidence of next events following COVID-19 onset, for the selected parametric models with and without censoring, compared to Aalen-Johansen nonparametric estimates to check

goodness of fit. By month of onset, comparing people with and without any comorbidities by age group (for men, excluding people under 45, care home residents and healthcare workers). Nonparametric estimates not shown where there are no observations in the category.

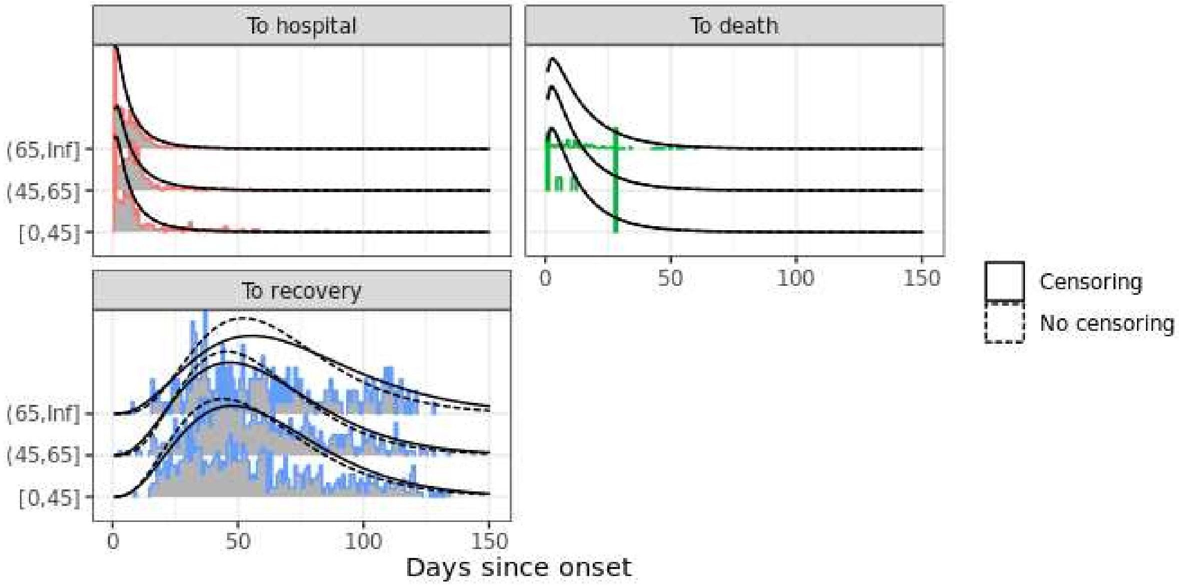

Appendix Figure 11. Illustration of fit of parametric models for times to three competing events following onset (hospital admission, death and recovery). Histograms of the observed times to events are compared with fitted probability densities from two alternative parametric models (accounting for or neglecting censored data), for the subgroup defined by female, no comorbidities, and not a healthcare worker or care home resident, and compared between three age groups

Combined hospital & community models

Appendix Figures 12 and 13 illustrate the goodness-of-fit of the parametric assumptions for the combined models that describe events following onset outside hospital and events following hospital admission. Predictions of cumulative incidence from the parametric model agree with nonparametric Aalen-Johansen estimates, for each event by subgroup.

Model with no covariates: goodness of fit

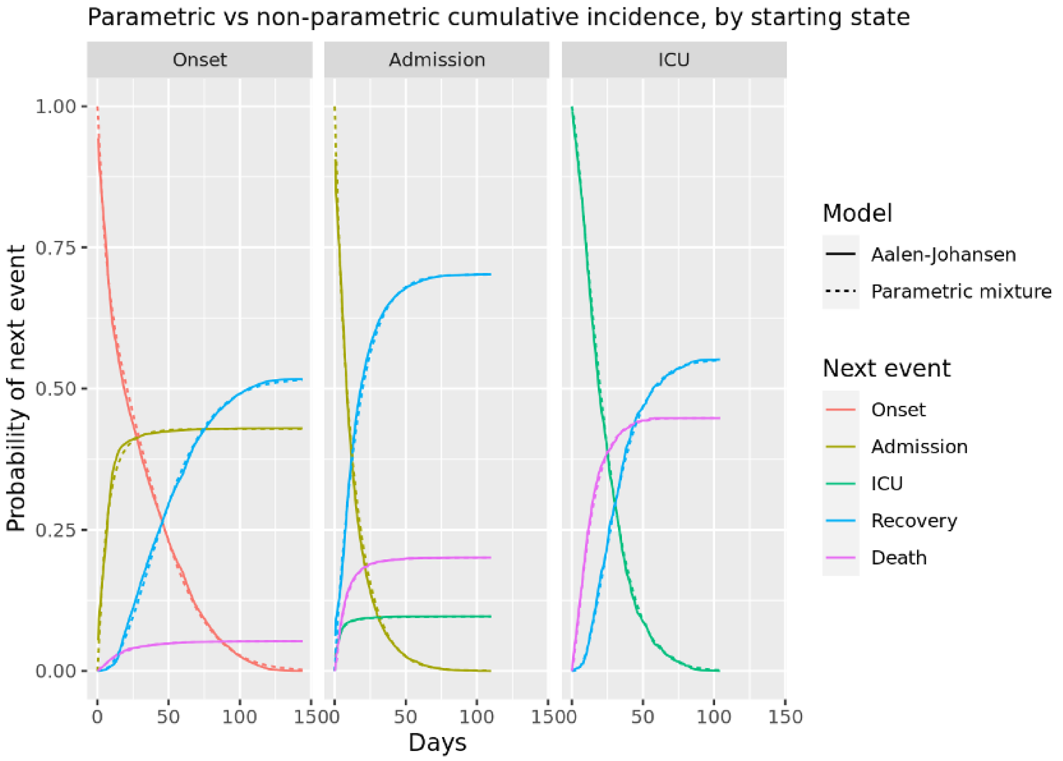

Appendix Figure 12. Predicted cumulative incidence of next events following COVID-19 onset, hospital admission or ICU admission, for the combined parametric model with no covariates, compared to Aalen-Johansen nonparametric estimates to check goodness of fit.

**Final event**	**Pathway**	**Mean Days**	**Lower**	**Upper**	**Median**	**Lower**	**Upper**	**2.5%ile**	**Lower**	**Upper**	**97.5%ile**	**Lower**	**Upper**
Death	Onset-Admission-Death	16.8	16.2	17.2	13.1	12.7	13.5	2.5	2.3	2.6	52.6	49.9	55.7
Death	Onset-Death	19.0	17.7	20.0	12.9	12.2	13.8	0.8	0.7	1.0	69.9	65.5	77.7
Recovery	Onset-Admission-Recovery	23.1	22.8	23.5	19.2	18.7	19.5	2.8	2.5	2.9	68.3	63.7	69.1
Death	Onset-Admission-ICU-Death	27.0	25.8	29.1	22.6	21.7	23.8	5.4	5.1	6.0	73.3	65.5	77.2
Recovery	Onset-Admission-ICU-	45.7	44.0	47.8	40.9	39.9	42.7	14.2	12.7	14.9	100.0	94.8	107.2

	Recovery												
Recovery	Onset-Recovery	49.5	49.1	50.1	46.0	44.3	45.9	9.7	9.4	10.6	115.3	111.8	117.2

Appendix Table 19. Estimated mean and quantiles of distribution of time from symptom onset to final event, by pathway and final event, from combined hospital/community model with no covariates.

Model with no covariates: pathway probabilities

The most common pathway is Onset-Recovery (estimated probability of taking this pathway 51.8% [51.2-52.3%]), followed by: Onset-Admission-Recovery (30.2% [29.7-30.7%]); Onset-Admission-Death (8.6% [8.3-9.0%]); Onset-Death (5.3% [5.0-5.5%]); Onset-Admission-ICU-Recovery (2.3% [2.1-2.5%]); and Onset-Admission-ICU-Death (1.9% [1.7-2.0%]).

Model accounting for age, gender and month of onset

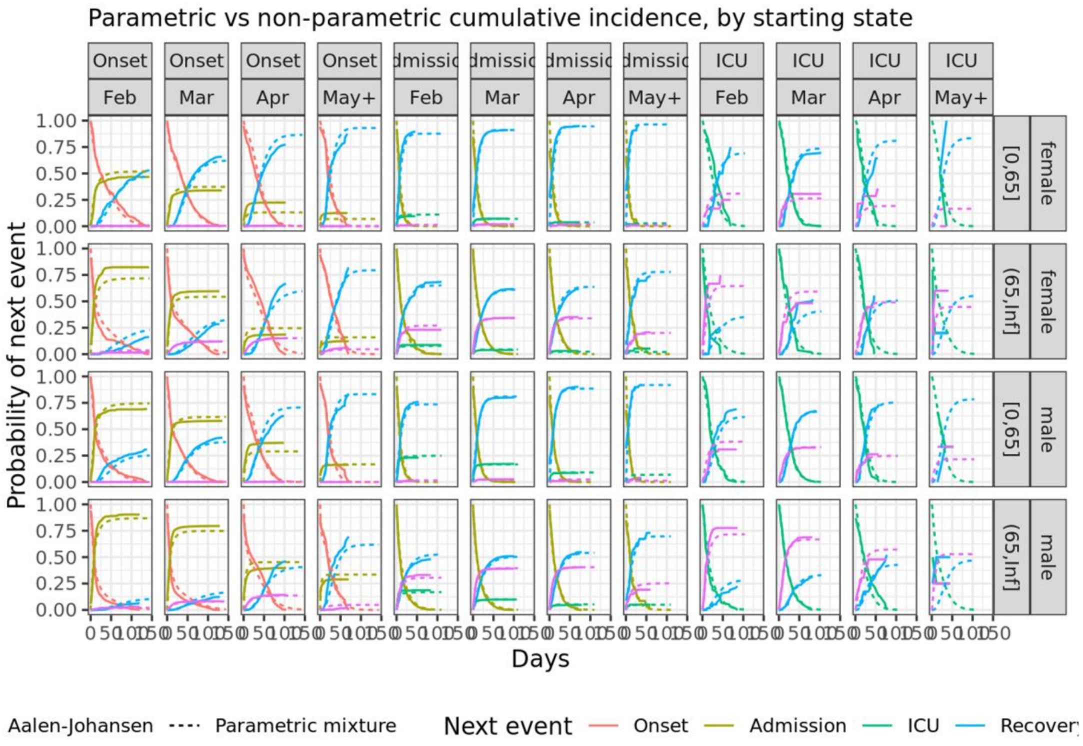

Appendix Figure 13. Predicted cumulative incidence of next events following COVID-19 onset, hospital admission or ICU admission, for the combined parametric model regressed on age, gender and month of onset, compared to Aalen-Johansen nonparametric estimates to check goodness of fit.

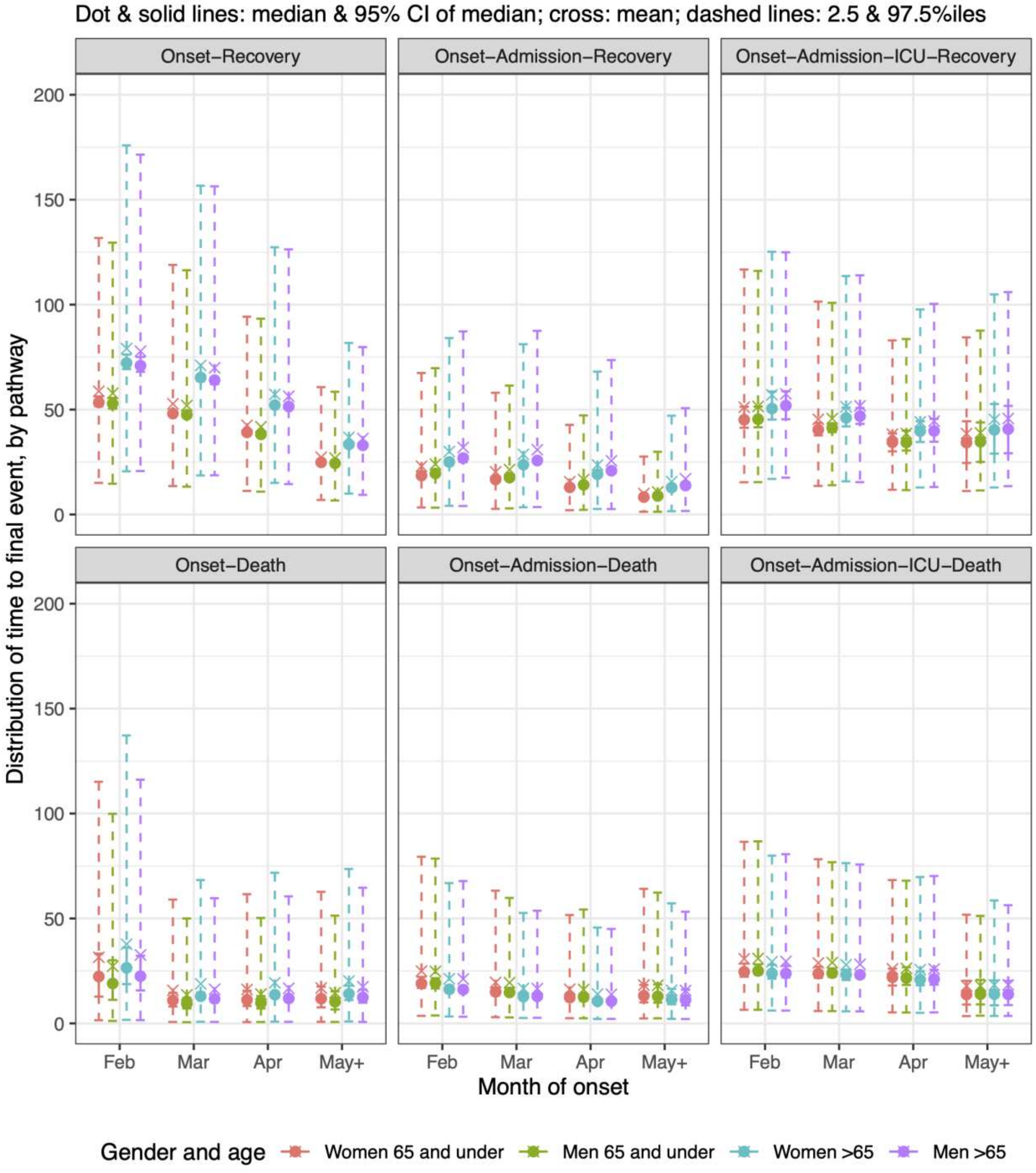

Appendix Figure 14. Estimated mean and quantiles of distribution of time from symptom onset to final event, by pathway, gender, age and month of symptom onset.

The estimated times from symptom onset to either confirmed recovery or death, averaged over pathway, by gender, age group and month of onset, are given in Appendix Figure 15. Younger age is associated with slightly shorter times to confirmed recovery and longer times to death. Men have slightly shorter median and mean times to confirmed recovery, but gender is not clearly associated with time to death. There is a suggestion of a decrease in mean and median time to death with calendar month, and also a decrease in heterogeneity in the distribution of time to death; though it is unclear whether case-mix in later months contributes to these changes. Similarly, the median and mean times to confirmed recovery do not vary much with calendar time, but there is a decrease in heterogeneity of times to confirmed recovery with month of onset.

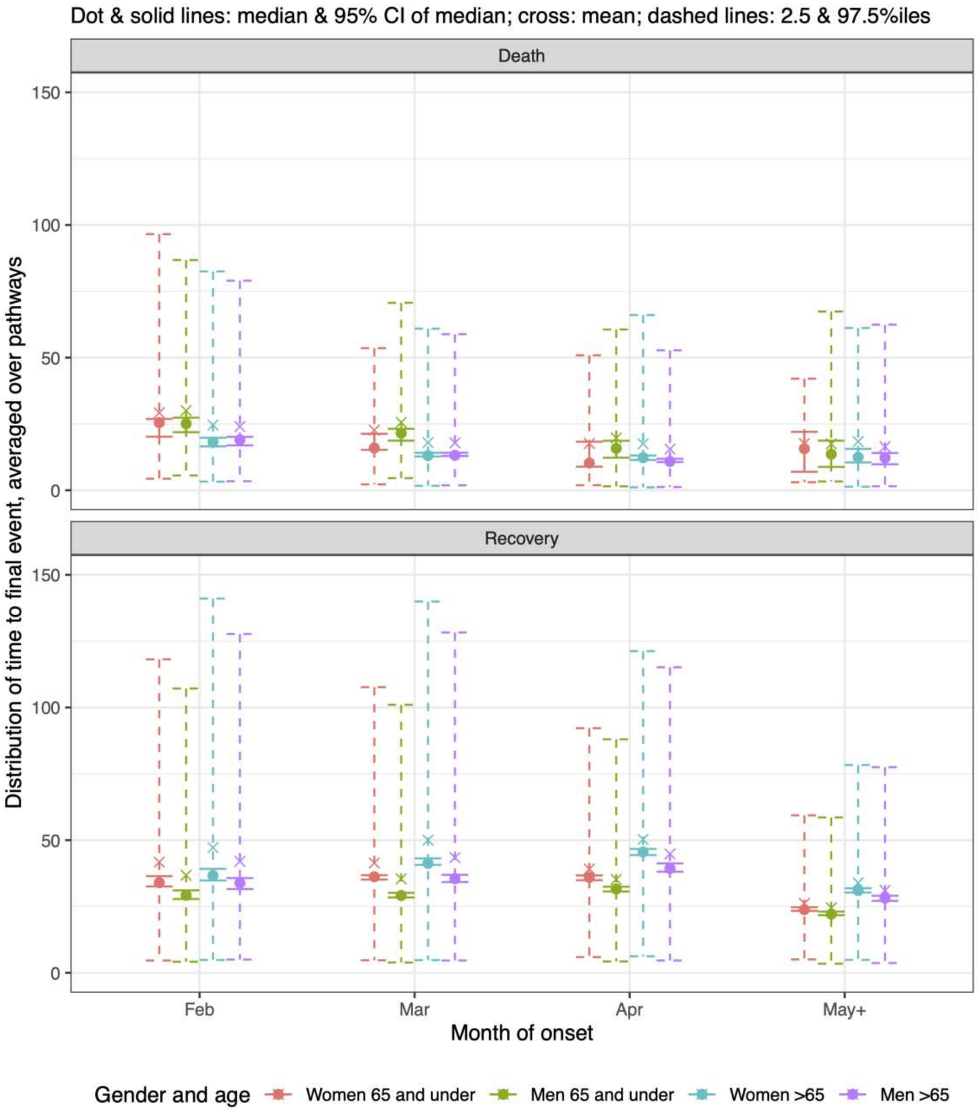

Appendix Figure15. Summaries of estimated distributions of times from symptom onset to death or confirmed recovery, averaged over pathways, by gender, age and month of onset.

References

Castillo, Gabriele Del, Francesca Grosso, Giuseppe Marano, Patrizia Boracchi, and Silvana Castaldi. 2020. "From Positive to Negative : A Time to Event Analysis in Regione Lombardia .," 4–9.

Cereda, Danilo, Antonio Federici, Angela Guarino, Grazia Serantoni, J. A. Bastiampillai, E. Gabrielli, F. Grimaccia, et al. 2020. "Development and First Application of an Audit System for Screening Programs Based on the PRECEDE-PROCEED Model: An Experience with Breast Cancer Screening in the Region of Lombardy (Italy)." *BMC Public Health* 20 (1): 1–11. https://doi.org/10.1186/s12889-020-09842-8.

Jackson, Christopher H. 2018. "Flexsurv: A Platform for Parametric Survival Modeling in R." *J Stat Softw* 70. https://doi.org/10.18637/jss.v070.i08.flexsurv.

Voinsky, Irena, Gabriele Baristaite, and David Gurwitz. 2020. "Effects of Age and Sex on Recovery from COVID-19: Analysis of 5769 Israeli Patients." *Journal of Infection* 81 (2): e102–3. https://doi.org/10.1016/j.jinf.2020.05.026.

Welfare Directorate of Regione Lombardia. 2020. "Regional Decree N° XI/3018; 30 March 2020." 2020. https://www.sicp.it/wp-content/uploads/2020/04/DGR-3018-Lombardia_gestione-emergenza-covid-in-RSA-e-RSD_30-mar-20_cprd.pdf.
